# Structural and functional characterisation of a stable, broad-specificity multimeric sialidase from the oral pathogen *Tannerella forsythia*

**DOI:** 10.1042/BCJ20220244

**Published:** 2022-09-06

**Authors:** Marianne J. Satur, Paulina A. Urbanowicz, Daniel I. R. Spencer, John Rafferty, Graham P. Stafford

**Affiliations:** 1 School of Clinical Dentistry, The University of Sheffield, 19 Claremont Crescent, Sheffield S10 2TA, U.K.; 2 Ludger Ltd., Culham Science Centre, Oxfordshire OX14 3EB, U.K.; 3 Department of Molecular Biology and Biotechnology, University of Sheffield, Firth Court, Western Bank, Sheffield S10 2TN, U.K.

**Keywords:** enzyme, periodontitis, sialic acid, sialidase

## Abstract

Sialidases are glycosyl hydrolase enzymes targeting the glycosidic bond between terminal sialic acids and underlying sugars. The NanH sialidase of *Tannerella forsythia*, one of the bacteria associated with severe periodontal disease plays a role in virulence. Here, we show that this broad-specificity enzyme (but higher affinity for α2,3 over α2,6 linked sialic acids) digests complex glycans but not those containing Neu5,9Ac. Furthermore, we show it to be a highly stable dimeric enzyme and present a thorough structural analysis of the native enzyme in its apo-form and in complex with a sialic acid analogue/ inhibitor (Oseltamivir). We also use non-catalytic (D237A) variant to characterise molecular interactions while in complex with the natural substrates 3- and 6-siallylactose. This dataset also reveals the NanH carbohydrate-binding module (CBM, CAZy CBM 93) has a novel fold made of antiparallel beta-strands. The catalytic domain structure contains novel features that include a non-prolyl cis-peptide and an uncommon arginine sidechain rotamer (R306) proximal to the active site. Via a mutagenesis programme, we identified key active site residues (D237, R212 and Y518) and probed the effects of mutation of residues in proximity to the glycosidic linkage within 2,3 and 2,6-linked substrates. These data revealed that mutagenesis of R306 and residues S235 and V236 adjacent to the acid–base catalyst D237 influence the linkage specificity preference of this bacterial sialidase, opening up possibilities for enzyme engineering for glycotechology applications and providing key structural information that for *in silico* design of specific inhibitors of this enzyme for the treatment of periodontitis.

## Introduction

Life at the host–pathogen interface places bacteria (and viruses) in a glycan-rich environment to which pathogens have adapted in several ways. One evolutionary development is the ability to bind to and harvest glycans from the glycoproteins at this interface [1,2]. Despite often being overlooked, the oral mucosal interface is rich in glycans with several oral bacteria either using terminal sugars as receptors or cleaving them for nutritional or immune modulatory purposes [1,3,4]. One of the most common terminal sugars on surface glycan chains in humans is sialic acid, a nonulosonic acid linked to underlying sugars via an alpha-glycosidic linkage between C2 of sialic acid and the C3 or C6 position of the next sugar with resultant α2,3 or α2,6 links. Unsurprisingly sialic acid is a key receptor for viral and bacterial pathogen adhesion [4–6], being important for influenza infection [7], as well as bacterial toxin attachment [8,9] and bacterial pathogen adhesion. In addition to sialic acid acting as a receptor, several bacterial species use sialic acid as a carbon and energy source or harvest and reprocess this sugar onto their cell surface as part of their lipopolysaccharide or capsular material [10–13]. In many cases, the first part of this process is the exogenous removal of sialic acid from the terminal glycan position using enzymes known as sialidases (or neuraminidases).

Bacterial sialidases are members of a group of glycoside hydrolases categorised as GH33 (CaZybase [14]). They utilise a retaining mechanism to hydrolyse the glycosidic bond between sialic acid and its underlying sugar via the action of active site residues that include a tyrosine nucleophile and an acid/base catalyst (often an aspartate residue) [15–17]. In addition, sialidases contain a suite of conserved residues that includes an arginine triad, which coordinates the carboxylate moiety of sialic acid (C1), a series of Asp-boxes (SxDxGxTW) that are believed to have a largely structural role, that are distributed around the six blades of a conserved beta-propeller structure, and a FRIP (phenylalanine–arginine–isoleucine–proline, where one of the Arginine residues is part of the catalytic triad) motif common in bacterial enzymes [18,19]. Finally, many bacterial sialidases also contain additional domains that are often involved in the binding of relevant sugars. These domains are known as carbohydrate-binding modules (CBM) [20], of which CBM32 and 40 are associated with sialidases and sialoglycan binding [20,21].

Among the oral microbiota, several bacterial species are known to produce sialidases. However, recent focus has been on the role of sialidases in a group of anaerobic bacteria known as the red-complex or keystone pathogens that are the most strongly associated with periodontitis [22,23]. All three of these organisms (*Treponema denticola*, *Porphyromonas gingivalis* and *Tannerella forsythia*) produce sialidases [4,24,25]. The sialidase from *T. forsythia*, an asaccharolytic oral bacterium resident in the sub-gingival plaque of humans [26], has been shown to have a role in biofilm growth, in human cell interaction and is able to strip sialic acid from human cells that is then processed via a dedicated transport and catabolism system that also contains a novel outer membrane TonB-dependent sialic acid permease [27–29]. This sialidase (NanH) is a secreted enzyme with a pH optimum of 5.5–6 but with good activity up to pH 7. It has the ability to cleave α2,3 and α2,6 linked sialic acids, with a preference for α2,3 over α2,6 linkages [30]. The C-terminal catalytic domain is preceded by a 160 amino acid N-terminal domain that is a CBM that our laboratory discovered is capable of binding various sialoglycans and non-sialylated derivatives but has no catalytic activity itself [30]. Sialidases seem to be important in the virulence of *T. forsythia* and other periodontal pathogens, mediating cell attachment invasion and biofilm formation with their inhibition presenting a potential novel treatment for this chronic and prevalent disease, which affects >750 million people globally. Periodontal disease is also associated with significant systemic sequelae such as rheumatoid arthritis, cardiovascular disease and possibly Alzheimer's [31–33]. Sialidases seem to be key in the ability to harvest mucin-based sialic acids by several members of the gut microflora, including *Bacteroidetes* members such as *Bacteroides fragilis* [34], *B*. *thetaiotaomicron* [35] and *Parabacteroides distastonis*, as well as unrelated *Bifidobacterium* spp. [36]. Notably, the sialidase of *Ruminococcus gnavus* represents a novel form of intramolecular *trans* sialidase that simultaneously cleaves and converts exclusively 2,3 linked sialic acid into a modified form that only it can utilise [37]. These bacteria also sit alongside enteric pathogens such as *Salmonella typhimurium* [38] and *Vibrio cholerae* [5] in all possessing sialidases important in their physiology and likely that of the gut microbiota in general.

In addition, glycoenzymes are currently used in the biopharmaceutical industry to aid in the profiling of recombinant protein products where glycosylation status is key to function, with one example being the current commercial use of sialate esterase from *T. forsythia* [39–41] and sialidases from anaerobes in the development of an IgG galactosylation assay [42]. Overall, it is thus imperative that we improve our knowledge of the structure and function of this class of enzymes to gain intimate knowledge of the molecular structure of the active site, how it binds ligands/drugs, and the identity of residues important for glycan co-ordination and specificity that will be key to future work on drug design and glycobiotechnology. Similarly, there is no information on the structure of the novel CBM in NanH from *T. forsythia*, and perhaps more surprisingly, no studies to date have investigated the binding of cleavable ‘native’ substrates by this family of well-conserved and widespread enzymes that are key to pathogenesis and life at the host–bacteria interface.

In this study, we set out on an in-depth structure–function characterisation of the glycan specificity, enzyme stability and three-dimensional structure of NanH in complex with a range of ligands, as well as conducting an extensive mutagenesis programme to probe the role of different residues in defining substrate specificity. During the study, we thus revealed the structure of the novel CBM and several unusual aspects of ligand co-ordination by the enzyme and identified residues that influence substrate specificity of sialidases and pave the way for future work.

## Results and discussion

### NanH is a stable enzyme with broad specificity for a range of human glycan structures

**Figure 1.**
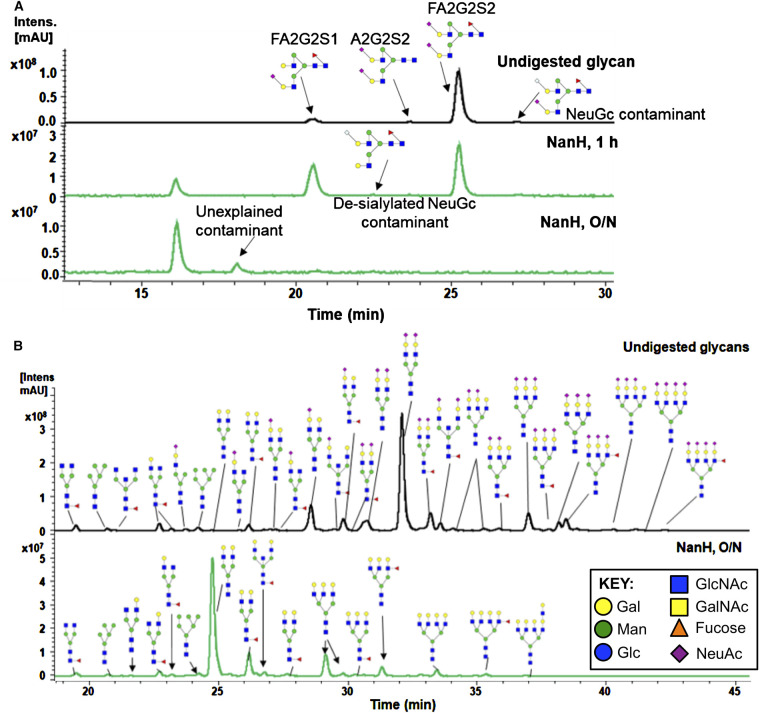
Ability of NanH to digest complex glycans. (**A**). LC-FLR traces of procainamide labelled sialidase digestions of FA2G2S2. Undigested (sialylated) FA2G2S2 (black trace). NanH 1h and O/N digests (green traces) are shown with known glycans. All symbols follow the Consortium for Functional Glycomics conventions and are shown in the Key: Gal-Galactose, Glc-Glucose, GalNAc-*N*-acetyl galactosamine, GlcNAc-*N*-acetyl glucosamine, Man-Mannose NeuAc-*N*-acetly-neuraminic/sialic acid. (**B**) LC-FLR traces of procainamide labelled human plasma N-glycans (black, control) and after overnight (O/N) incubation with NanH (0.1 mg ml^−1^).

Our previous work highlighted that NanH is a GH33 gamily enzyme and indicated the presence of a novel CBM that bound sialloglycans but also a preference for α2,3- over α2,6-linkages (∼2-fold higher efficiency (*k*_cat_*/K_M_*), see Figure 8B) and the ability of NanH to remove sialic acid from the surface of human oral epithelial cells [30]. Here, we probed in detail the preference of NanH for a range of simple and complex sialic acid-containing glycans using a combination of HPLC and mass spectrometry. These data revealed that NanH cleaved sialic acid from the host-relevant FA2G2S2 (also known as A2F) glycan (Figure 1A), a biantennary glycan where both branches terminate with a sialic acid and which is present on many secreted glycoproteins including IgG. Notably, other peaks were observed in the chromatogram for our FA2G2S2 preparation, which are likely to correspond to differently sialylated versions of the FA2G2S2 glycan and they also appeared to be desialylated. These other glycans included a Glycolyl-neuraminic acid (NeuGc) containing version, indicating the ability of *Tannerella* NanH to target these glycans and which builds on our previous observation that *T. forsythia* can utilise NeuGc substrates for growth [27].

**Figure 2.**
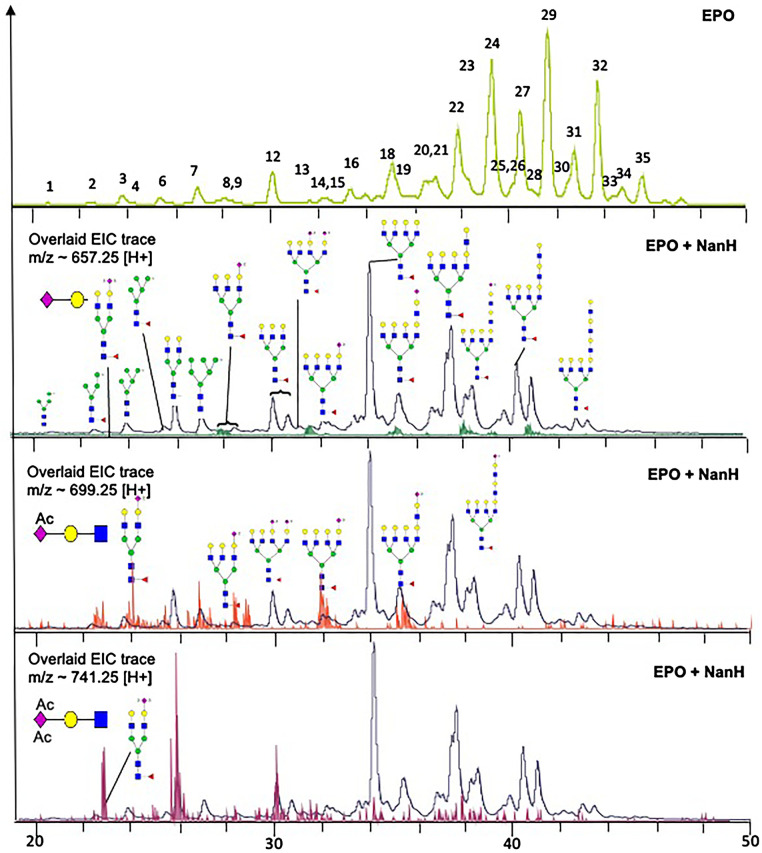
Effect of Neu5,9Ac glycans on digestion with NanH. N-glycan profile of erythropoietin N-glycans before (top panel, see Supplementary Table S1 for peak assignments) and after (lower panels) incubation with NanH (0.1 mg ml^−1^ o/n) overlaid with M/S traces for Neu5Ac (green), Neu5Ac-Mono-OAc (red) and Di-OAC (purple). Peak numbers in the upper panel are assigned according to Supplementary Table S1 and all symbols as shown in Figure 1.

We then tested the desialylation of glycans derived from more complex host-relevant substrates. Firstly, since it has some similarity to the Gingival crevicular fluid which bathes biofilm in periodontal plaque we analysed the release of procainamide labelled N-glycans from human plasma [39]. This data revealed that NanH is able to cleave sialic acid from the termini of a diverse range of glycans that includes fully and partially sialylated versions of bi-, tri- and tetra-antennary branched glycans, as well as those with branching fucose residues, (Figure 1B). Notably, this broad-specificity enzyme seemed to remove all sialic acid residues present in plasma N-glycans. In contrast, our own previous work had revealed that NanH was likely unable to cleave sialic acids that were further modified with O-acetyl groups at position C9, C8, C7 or C4 and present in mucins without the assistance of a companion sialate esterase (NanS) [40]. Therefore, we analysed the NanH digestion profile of the well-characterised glycoprotein erythropoietin (EPO), which we had previously shown contains multiple bi-, tri- and tetra-branched glycans, with α2–3 and α2–6 sialic acid linkages, with significant levels of mono or di-O-acetylated species (Neu5,9Ac, and tri-acetylated Neu5,8,9Ac) [40,41]. The EPO was digested with NanH, N-glycans were labelled and underwent UHPLC-FLD-ESI-MS/MS (Figure 2). Following digestion with NanH, we observed a marked change in the chromatogram with all peaks lost that contained sialic acid except for those that contained sialic acids that had one or two extra O-acetyl groups present (Figure 2, 2nd panel). Specifically, these analyses showed that the extracted ion chromatogram (EIC) corresponding to the MS2 ion for Neu5Ac trisaccharide (GlcNAc-Gal-Neu5Ac, *m/z* = ∼657.25) was not detected above background levels (green EIC trace), while EIC corresponding to di- and tri-acetylated sialic acid trisaccharides (GlcNAc-Gal-Neu5,9Ac and GlcNAc-Gal-Neu5,8,9Ac, *m/z* = ∼699.25 and ∼741.26; red and purple traces — lower two panels) were detected in high abundance (high peak intensity) in the NanH-treated sample. These data show definitively that sialic acids with O-acetylated groups protect the sialolglycan from glycosidic cleavage by NanH sialidase (for details of peak designations in the untreated EPO, see Supplementary Table S1). As highlighted in our previous work, this has implications in the oral cavity where several proteins, including cell surface proteins as well as salivary mucins are likely to contain this acetylation modification. Indeed our own work indicates that NanH is able to do this as *T. forsythia* can grow on mucin substrates and release sialic acids from them (4,52).

Finally, given that NanH is a secreted virulence factor that may be a useful broad-specificity enzyme for biotechnology we conducted a series of experiments testing its stability. The data show that even after prolonged incubation at 25°C (RT) and 37°C for 48 h as well as five rounds of freeze–thaw (FT) cycling, the enzyme retained nearly 100% activity, while unsurprisingly after incubation at 55°C activity was lost, presumably because of denaturation of the enzyme (Supplementary Figure S1A–D). As a control, we also showed that there was no background mannosidase activity (Supplementary Figure S1E). In parallel to these studies, we also stored the enzyme after storage for prolonged periods of up to 9 months (Supplementary Figure S2A–D) and accelerated degradation (incubation at 24–48 h at 37 and 42°C) conditions (Supplementary Figure S2E) to establish its potential usability as a commercial product. Under these conditions, NanH retained full activity at 37°C for 48 h and was still 60–70% active after 9 months storage under a range of conditions (Supplementary Figure S2A–D).

Taken together these data provide direct evidence that NanH is a highly stable enzyme able to persist at human body temperature for extended periods (over 24 h), which would provide clear physiological advantages to *T. forsythia* and the oral microbiome in terms of glycan harvesting potential. Our data also highlight and define conditions for the retention of activity at −20°C and repeated FT cycles, which is an essential element if it is to be used as a laboratory reagent. This stability means that this type of extracellular glycosidase might have the potential for manufacture and use in enzyme-based glycan analytic pathways [43], as has been seen for the *T. forsythia* sialate esterase, NanS [40]. Alternatively, sialidases have been proposed as therapeutics for cancer treatment to combat hypersialylation, for which their broad specificity and notable stability might make NanH and NanS ideally suited [44].

### Determination of structural characteristics of NanH sialidase

**Figure 3.**
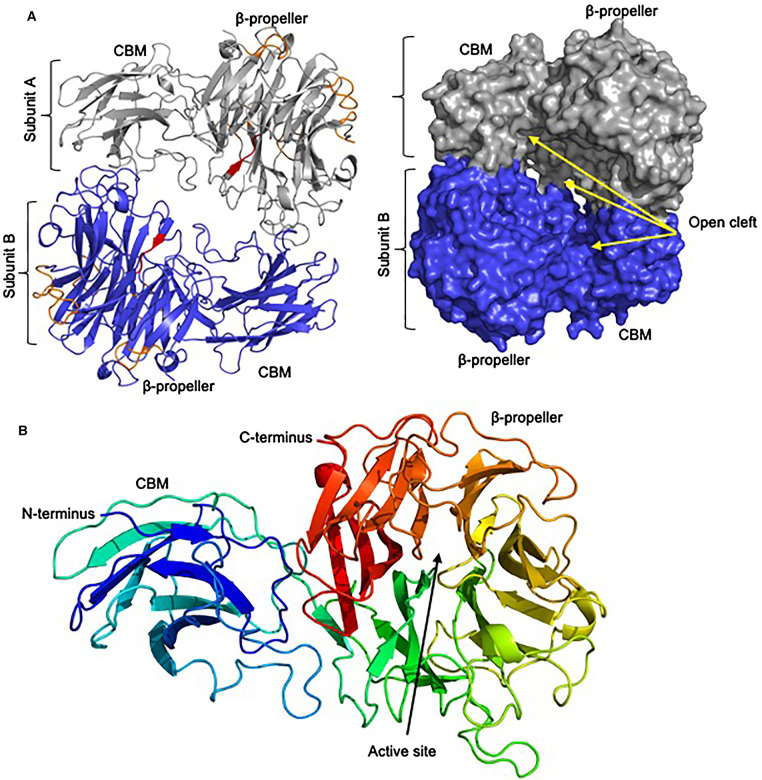
Crystal model of NanH-apo. (**A**) Each subunit (A in grey and B in blue) comprises a Beta-propellor (β-propellor) containing the active site and FRIP motif (red), Asp-boxes (orange) and an N-terminal Carbohydrate-binding module (CBM). Shown in cartoon and surface visualisation from Pymol with open cleft between subunits labelled. (**B**) Subunit A of NanH-apo, highlighting individual blades of the β-propeller domain in rainbow representation and with active site labelled.

To enable structure–function investigations of NanH, we set out to establish a structural model of this enzyme. Using our established protocol for producing highly purified and concentrated enzyme [30] (Supplementary Figure S3), NanH was screened in sitting-drop crystallisation trials, with conditions containing protein in 0.1 M HEPES pH 6.0, 7% w/v PEG 6000 producing robust crystals. The first crystal structure of NanH was solved to a resolution of 2.0 A, using a related sialidase (PDB: 4BBW, 67% amino acid identity) as a model in the molecular replacement. The data collection and refinement statistics are shown in Table 1. The model consisted of 519 residues (34–552) in each subunit (Figure 3, Supplementary Figure S4) and showed that there were two copies of NanH in the asymmetric unit.

**Table 1.** Data collection and refinement statistics for NanH and NanH variants

Parameters	NanH-apo	NanH-HEPES	NanH-Oseltamivir	NanH-D237A-apo	NanH-D237A-3SL	NanH-D237A-6SL
Crystallisation conditions	0.01 M HEPES pH 6.0, 7% w/v PEG 6000	0.1 M HEPES pH 6.0, 7% w/v PEG 6000	Same as NanH-apo, plus 5mM oseltamivir soaking step (30s)	0.01 M HEPEs pH 7.0, 10% w/v PEG 6000	Same as D237A-apo with soaking in 5 mM 3-SL (30 s)	Same as D237A-apo with soaking in 5 mM 6-SL (30 s)
Space group	P 41 21 2	P 41 21 2	P 41 21 2	P 41 21 2	P 41 21 2	P 41 21 2
Unit cell parameters:						
* a* (Å)	79.9	79.7	79.5	80.1	80.1	79.9
* b* (Å)	79.9	79.7	79.5	80.1	80.1	79.9
* c* (Å)	349.3	349.5	348.9	349.9	349.8	349.6
Resolution range (Å)	1.66 (1.70–1.66)	1.97 (2.02–1.97)	1.92 (1.97–1.92)	2.11 (2.16–2.11)	2.06 (2.11–2.06)	1.90 (1.95–1.90)
No. asymmetric unit	2	2	2	2	2	2
*R*_merge_ (all I^+^ and I^−^)	0.17 (0.89)	0.06 (0.90)	0.15 (1.02)	0.13 (2.27)	0.16 (2.91)	0.14 (2.70)
*R*_pim_ (all I^+^ and I^−^)	0.05 (0.34)	0.02 (0.49)	0.04 (0.30)	0.04 (0.66)	0.03 (0.58)	0.03 (0.59)
Mean ((I)/sd (I))	6.9 (0.9)	21.8 (1.4)	10.6 (1.9)	11.4 (1.0)	14.8 (1.5)	14.4 (1.3)
Completeness (%)	100.0 (100.)	99.4 (97.4)	100.0 (100.0)	100.0 (100.0)	100.0 (100.0)	100.0 (100.0)
Multiplicity	12.1 (7.9)	10.5 (4.0)	12.6 (12.2)	12.8 (12.8)	25.6 (26.1)	25.3 (21.8)
*R*-factor (%)	0.23	0.21	0.31	0.25	0.23	0.26
*R*_free_ (%)	0.27	0.27	0.36	0.30	0.29	0.31
No. refined atoms: protein/ligands/water	8078/40/704	8052/30/427	8048/20/292	8043/—/124	8050/64/290	8050/68/398
Average B-factors: protein/ligands/water	32/48/34	49/54/44	43/34/33	58/—/50	56/71/48	52/59/43
Ramachandran plot:						
In preferred regions (%)	94.7	93.6	92.7	92.9	93.5	93.0
In allowed regions (%)	3.9	5.2	5.9	5.8	5.1	5.7
Outliers (%)	1.4	1.2	1.5	1.3	1.4	1.3
PDB entry code	7QYP	7QZ3	7QY9	7QYJ	7QY8	7QXO

Numbers in parentheses are statistics for the outer shell.

**Figure 4.**
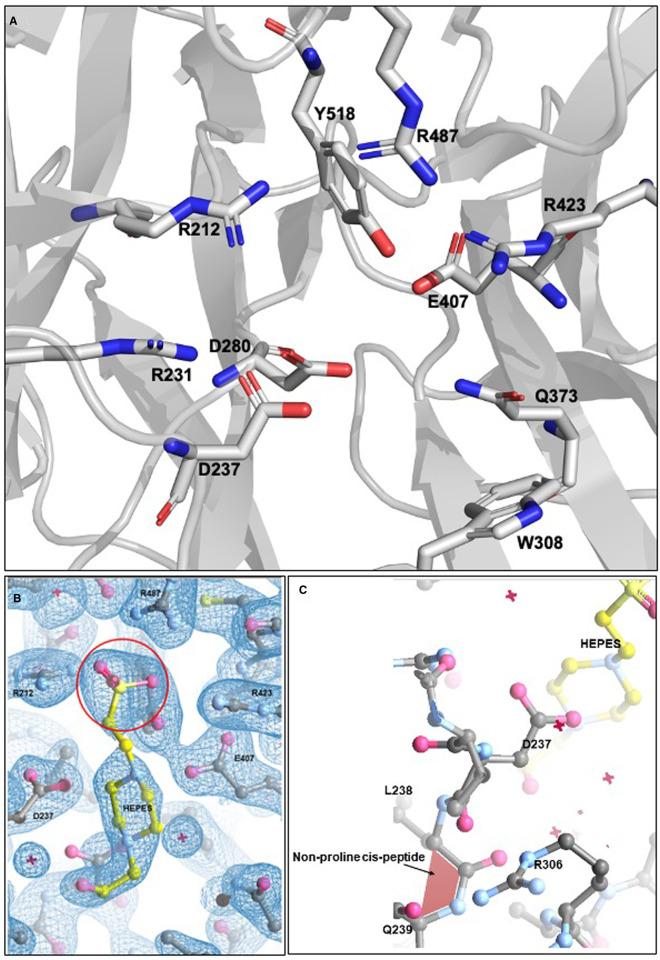
Detailed view of the NanH active site. (**A**) Shows the catalytic arginine triad of R212, R487 and R423 alongside the catalytic nucleophile Y518, D237 and E407 as well as other key residues. (**B**) Displays the bound HEPES molecule in yellow with the –COOH group circled. (**C**) Location of the non-proline cis-peptide at Q239 in relation to the bound HEPES.

The NanH catalytic domain (residues 197–552) folds into a characteristic 6-bladed β-propeller catalytic domain, with each blade being composed of 3–4 antiparallel β-strands (Figure 3). The location of the active site in NanH is highlighted by the presence of the conserved catalytic residues at the centre of the propeller, where D237 is the conserved aspartate, which is believed to be involved in the catalytic mechanism along with Y518 and E407, which are likely the nucleophile and acid–base residues, based on structural homology (Figure 4). Surprisingly, in our first crystal form, the active site was occupied by a molecule of HEPES (Figure 4B, shown in yellow), with its sulfate group occupying the predicted position of the sialic acid carboxyl group expected from its location in other structures and being co-ordinated by R212, R423 and R487 that form an arginine triad. (Figure 4A,B). A lower concentration of HEPES in the crystallisation buffer of 0.01 M did result in subsequent datasets and maps to which a model was fitted that lacked the HEPES molecule but otherwise had identical features (Figure 4A), implying that binding of HEPES did not cause changes in conformation. It is also notable that the NanH model shows a non-proline cis-peptide between residue L238 and Q239 (Figure 4C). This non-proline cis-peptide is conserved in the structures of the sialidase from the closely related *P. distastonis* but not in BTSA of *B. thetaiotaomicron* [35]. This proline likely helps to establish the structure of the binding pocket and may co-ordinates a solvent molecule positioned potentially to interact with the sialic acid moiety of the substrate potentially to position the arginine in proximity to the carboxylate group. In this region of the model adjacent to the cis-peptide in space, we also see an uncommon arginine rotamer (observed in other protein structures with ≤1% frequency) for R306 (Figure 4C). This residue is present on a loop located in close proximity to the active site and is only conserved in sialidases with high sequence identity with NanH, such as BTSA and the sialidase from *P. distasonis* (PDB 4FJ6).

**Figure 5.**
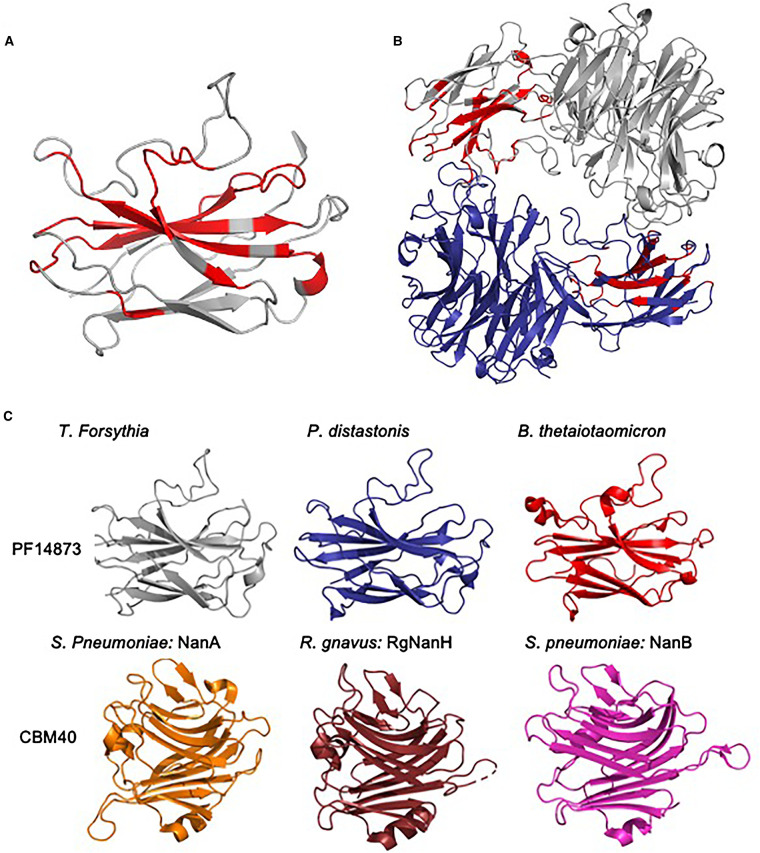
The CBM of *T.forsythia* forms a novel CBM family. CBM (**A**) and entire enzyme (**B**) with conserved putative binding site residues (as shown in Supplementary Figure S5) highlighted in red. (**C**) *T. forsythia* NanH alongside equivalent predicted CBM from the closely related *P. distasonis* (PDB: 4FJ6) and BTSA from *B. thetaiotaomicron* (PDB: 4BBW) and the CBM40 sialic acid binding CBM from *S. pneumoniae* (PDB: 4ZXK), RgNanH from *R. gnavus* (PDB: 6ER2) and NanB from *S. pneumoniae* (PDB: 2VW0).

In addition to determining the structure of the catalytic domain, we also resolved a model for the N-terminal domain (residues 34–196, i.e. 150 amino acids), which we previously showed experimentally to be a CBM with affinity for sialoglycans [30]. The data here reveal that the CBM is composed of 4 antiparallel β-strands that form the base of an open cleft. The base of this CBM cleft is rich in highly conserved residues (regions highlighted in red in Figure 5 and shown in Supplementary Figure S5). Further analysis of an amino acid sequence alignment of this group of enzymes reveals this CBM exists within several confirmed and predicted sialidases across a range of *Bacteroidetes* — including gut *Bacteroides* spp., the vaginal *Prevotella bivia* and the soil organism *Pedobacterium heparinus* (Supplementary Figure S5). The cluster of conserved residues in this CBM contains arginine residues which may have relevance in co-ordination of the –COOH moiety of sialic acid as is the case for the CBM40 domain of *R. gnavus* [45], suggesting this region may bind the glycan ligand — although we have no evidence at present. As noted in our previous work, BLAST and PFAM database searches using the sequence of the NanH-CBM did not reveal homology with other sialic acid binding CBM, such as CBM40. Crystal structures of the CBM40 domains of sialidases from *S. pneumoniae* and *R. gnavus* are shown in Figure 5 and are noticeably larger than the NanH-CBM from *T. forsythia* and other family members (i.e. approximately 200 residues vs the 150 residues of the NanH CBM). The NanH-CBM also has a β-sandwich style fold with a central cleft; however, the structure is more open. The structures of CBM40 domains from *S. pneumoniae* and *R. gnavus* have been determined bound to sialyllactose [21,46], which was located in the equivalent regions to that of the conserved residues on the upper beta sheet of the NanH-CBM. We propose that the NanH CBM is a member of a novel family of CBM- which has been assigned CBM93 in the CaZy database [14]. CBM93 is annotated as PF14873 in the Pfam database and is often associated with sialidases, but also with GH18 hydrolases and in proteins containing laminin_G_3 lectin domains that are associated with many types of Glycosyl hydrolases. Thus we propose that this distinctive *T. forsythia* NanH CBM most likely acts to position the enzyme active site for cleavage of multiple sialylated residues on the bi- and tri-antennary substrates encountered by NanH in the oral cavity.

In the asymmetric unit of the crystal structure there are two copies of the NanH monomer and analysis of their packing using the PISA software [47] also suggests that they form a dimer (Supplementary Figure S6). The overall structure of this dimer reveals a two-fold symmetric arrangement with each catalytic domain facing a CBM domain between which there is an open cleft region (Supplementary Figure S6). Furthermore, the superimposition of the monomers using the SUPERPOSE program indicated the subunits are structurally identical (Supplementary Table S2). Despite the lack of kinetic co-operativity, the interface between these subunits is significant, with an interface area of 1575 Å^2^, equating to 8.2% of the total surface area (∼19 000 Å^2^) and a theoretical Δ*G*^diss^ of 10.1 kcal/mol. In the structural model, this interface seems to comprise around 47 residues stabilised by 30 hydrogen bonding events as well as a salt-bridge between D318 of one subunit and K64 of the other (Supplementary Figure S6A,B). To investigate if this was a crystallisation artefact or if NanH existed as a dimer in the solution we analysed it in solution by HPLC-SecMALs, which revealed that under the conditions tested it exists largely as a dimer in solution (90%) and with some indication of a possible minor tetrameric form (10%) (Supplementary Figure S7). Searching the PDB database reveals the closest structural homologues to NanH are BTSA and a sialidase from *Parabacteroides distanosis* (PDB: 4FJ6, 72% amino acid similarity). While BTSA is deposited as having a monomeric biological assembly, there is a clearly similar dimeric organisation of symmetry-related subunits to that seen for NanH, and in the *P. distanosis* sialidase model, which is assigned as being a biological tetramer. This is confirmed by analysis with the PISA program, which assigns the BTSA interface a Complex Formation Significance Score of 1.0 (on a scale of 0–1). All three enzymes display a high level of structural and sequence conservation across their dimer interfaces (1548 Å^2^, 1578 Å^2^ and 1440 Å^2^ of buried surface area for BTSA, NanH and *P. distanosis* sialidase, ∼90% sequence identity of interface residues) but the salt-bridge in the interface observed in NanH (K64:D318) is maintained only in the *P. distanosis* enzyme (K50-D306). Notably, NanH seems to be able to exist as a dimer or tetramer, but we have no data indicating whether stability or catalytic efficiency are modulated by this multimerization.

### Determination of ligand binding properties of NanH

**Figure 6.**
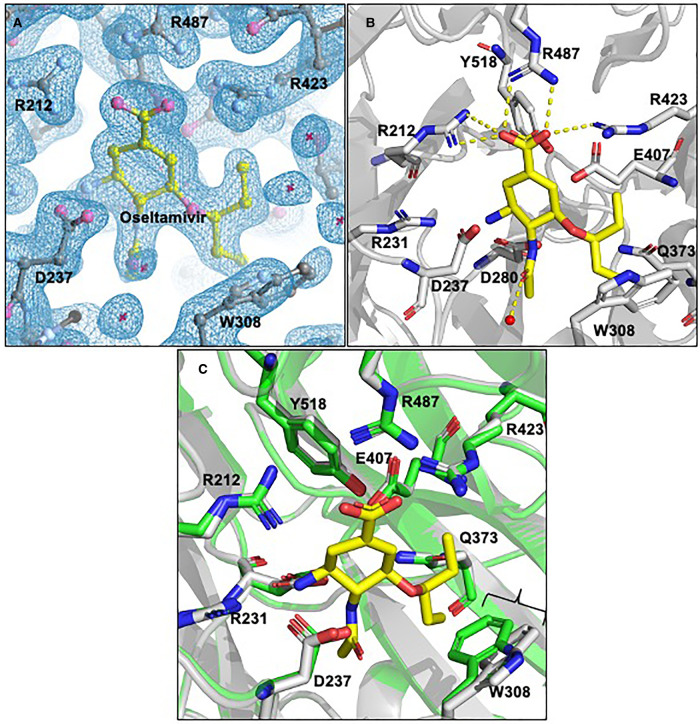
NanH active site bound in complex with the inhibitor oseltamivir. (**A**) Oseltamivir (yellow) was modelled into the electron density (blue) of the *F*_0_ − *F*_C_ double difference map at an rmsd of 1.04. (**B**) NanH sialidase in cartoon representation (grey), with the catalytic residues (grey sticks) and oseltamivir (yellow sticks). A single water molecule is highlighted as a red sphere and the yellow dashed lines represent the hydrogen bonding network; (**C**) Overlay of NanH-apo (green carbons) with NanH-oseltamivir (grey carbons) illustrating the movement of W308 (highlighted by bracket) upon the binding of oseltamivir (yellow) to the NanH active site.

As outlined in our previous work and that of others [6,24,27,30]; the sialic acid analogue inhibitors zanamavir, oseltamivir and DANA are able to inhibit whole cell sialidase activity of *T. forsythia*. After establishing the IC50 of these inhibitors with purified NanH (Supplementary Figure S8), we attempted co-crystallisation and crystal soaking with DANA and Oseltamivir since both had IC50 in the μM range. While we were unable to identify DANA in any crystal models, we could solve a model at 1.9 Å with Oseltamivir in the active site after soaking it into the NanH-apo crystals at a concentration of 5 mM (Figure 6A,B). The electron density for the oseltamivir is notably poorer in one subunit and could not be modelled reliably, implying a lower occupancy of the site, although the reason for this is not clear as access appears equivalent and the local protein residues adopt the same conformations. In this model, Oseltamivir is orientated in the active site via its negatively charged carboxyl group, which is held equatorial to the cyclohexene ring through hydrogen bonding interactions with the guanidiniums of the arginine triad (Figure 6A,B). The cyclohexene ring itself is sitting in a distorted boat conformation caused by the carboxyl group taking a pseudoequatorial position upon the binding of the arginine triad. The amino group of oseltamivir (position C9, Figure 6) interacts with the carboxylates of D237 and D280 while the *N*-acetyl tail sits in a hydrophobic pocket with a hydrogen bond forming between the amide of the *N*-acetyl and the carboxylate of D280. Comparison of the active site conformation between the NanH-apo and oseltamivir bound form shows that all residues have identical conformations except for the sidechain of W308 which is displaced by the pentyl ether group of oseltamivir, with an average displacement for the aromatic rings of the tryptophan of 1.4 Å (Figure 6C). There are no other major differences in the structure of the protein backbone or sidechains compared with NanH-apo.

Next, we set out to determine how NanH might co-ordinate a native ligand such as might be present on a cell surface glycoprotein. However, the wild-type NanH enzyme would turn over any substrate containing sialic acid and monomeric sialic acid does not bind NanH. So to achieve a substrate–enzyme complex, we produced an inactive variant that would retain the ability to bind substrate but without glycosidic activity. Residue D237 was selected for mutagenesis because it is a highly conserved aspartate, which when mutated in other sialidase enzymes (including Influenza A neuraminidase [7]), loses catalytic activity but retains binding capacity. We mutated D237 to alanine, producing an enzyme that was fully soluble but inactive (Figure 8B,C). We also showed that it still retained the ability to bind the oligosaccharide substrates 3-SL (*K_D_* 304.8 ± 91.5 µM) and 6-SL (*K_D_* 133.5 ± 48.3 µM) with comparable affinity to the WT enzyme using tryptophan quenching experiments. [30] (Supplementary Figure S9). After these experiments, we chose to use D237A in further crystallography experiments as structural predictions postulated it would not affect the position of surrounding residues. Using this mutant we produced crystals and determined structures for the NanH-D237A-apo protein (1.7 Å) and complexes with 3-SL (2.06 Å) and 6-SL (1.90 Å). Again these models contained two subunits in the asymmetric unit, while their overall structures were nearly identical with the wild-type NanH-apo forms in terms of backbone structure and dimeric interfaces except for the change in electron density for the D237A mutation (see Supplementary Table S1 for xyz displacement data).

**Figure 7.**
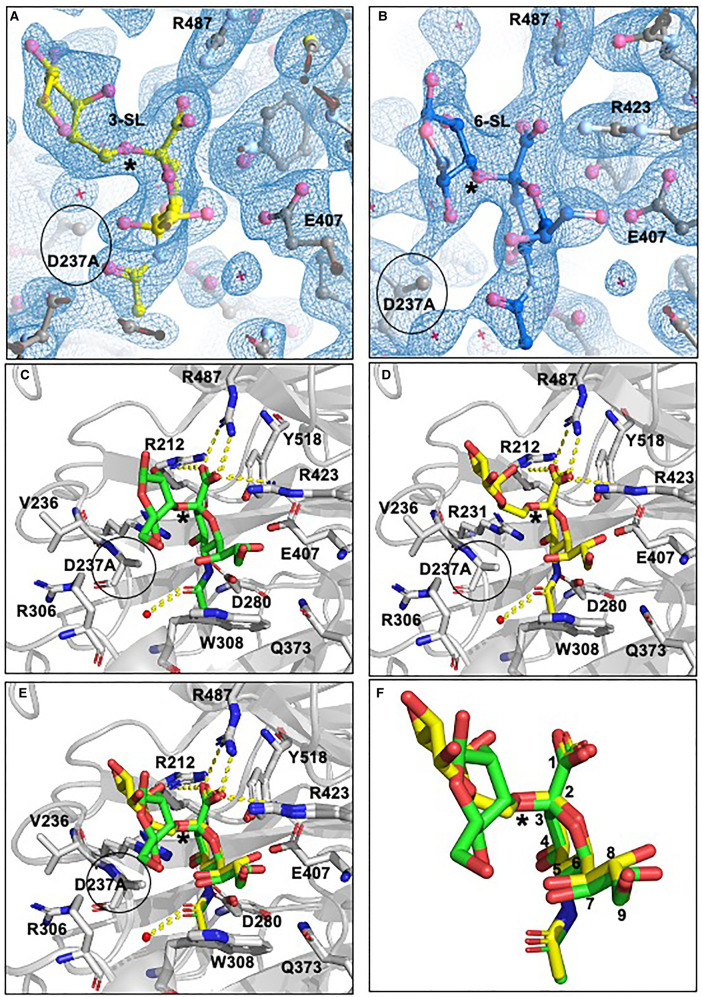
NanH-D237A bound in complex with 3- and 6-SL. (**A**) 3-SL (yellow) was modelled into the electron density (blue) of the 2*F*_0_ − *F*_C_ double difference map at an RMSD of 1.02. (**B**) 6-SL (dark blue) was modelled into the electron density (blue) of the 2*F*_0_ − *F*_C_ double difference map at an RMSD of 0.99. (**C**) NanH-D237A (grey cartoon) in complex with 3-SL (green sticks). (**D**) NanH-D237A in complex with 6-SL (yellow sticks). (**E**) NanH-D237A in complex with 3-SL (green sticks) overlaid with NanH-D237A in complex with 6-SL (yellow sticks). (**F**) Overlay of 3-SL (green sticks) and 6-SL (yellow sticks) isolated from the protein backbone (carbon atoms numbered). In all panels, the NanH-D237A protein is shown in grey in cartoon representation with all the key residues around the catalytic centre added in stick representation, and hydrogen bonding (yellow dashes) shown. D237A is circled in all panels while the glycosidic linkage is highlighted with an asterisk.

As might be expected, the terminal sialic acid of 3-SL and 6-SL are positioned in the active site of NanH-D237A with their carboxyl-groups (C1) co-ordinated by a hydrogen bonding network with the arginine triad residues (R423, R487 and R212) and the *N*-acetyl group hydrogen bonds with D280 in a similar manner to that seen in the NanH-oseltamivir model (Figure 7). The pyranose ring sits in a distorted boat conformation, while as a result of the D237A mutation, the C4-OH group makes contact with R231. Of note here, when the 3-SL and 6-SL structural models are overlaid, the sialic acid portion of 6-SL occupies a near identical space to that of 3-SL with no substantial differences (≥0.1 Å) between the hydrogen bond lengths connecting the 3-SL and 6-SL terminal sialic acids to the conserved catalytic residues (Figure 7). In these models, the non-proline cis-peptide is still present and R306 adopts the same rotamer form as in all the previous NanH models. Of note, there is no displacement of W308 in the 3-SL or 6-SL models in contrast with the NanH-oseltamivir model. The oseltamivir-dependent distortion may be attributed to its competitive inhibitory mechanism where it binds into the active site, but also suggests this region may be a target for the introduction of further modifications of oseltamivir in the design of a new inhibitor molecule.

**Figure 8.**
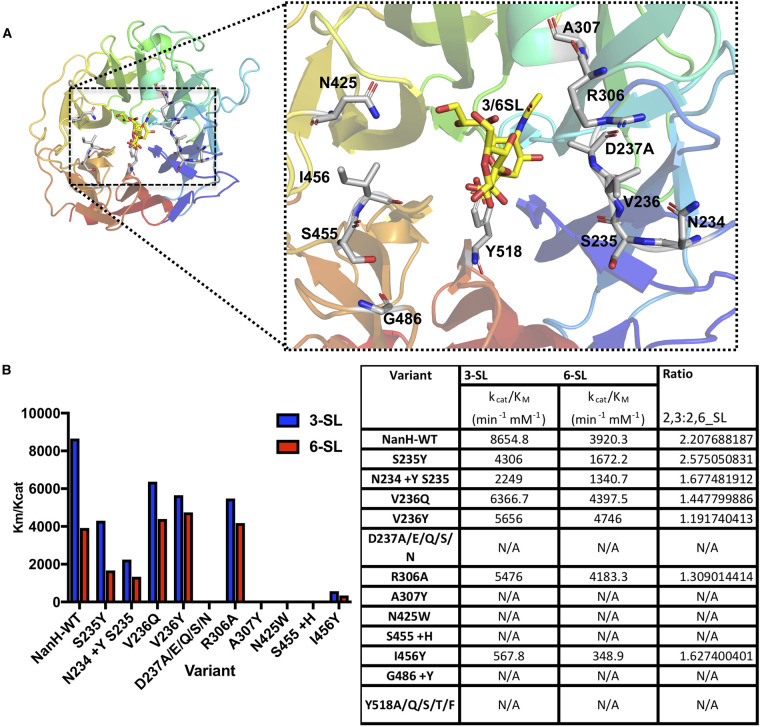
Structure–function mutagenesis of NanH. (**A**) Illustration of the catalytic domain in isolation showing the location of residues targeted by mutagenesis in relation to bound 3-SL or 6-SL. (**B**) Kinetic parameters of NanH and variants with 3-SL or 6-SL as assayed by TBA assay. The *k*_cat_/*K_M_* (efficiency) values are presented in the bar-chart on the left and in tabular form on the right, alongside the ratio of 3-SL : 6-SL. N/A indicates non-active.

Perhaps unsurprisingly the glycosidic linkage between C2 of sialic acid and the underlying galactose is in a near identical position in 3D space for both the 3-SL and 6-SL models, a phenomenon also seen in other proteins — such as Influenza A (HA) — the only other sialidase structures with these substrates in the active site [7]. This indicates that NanH can still present the glycosidic linkage in close enough proximity to the Tyr/Glu (Y518/E407) nucleophilic pair which is thought to be key to catalysis (Figure 7). As can be seen in Figure 7, the position of galactose could be modelled, showing that its position in the 3-SL and 6-SL complex structures is not identical. The galactose of the 3-SL complex is perpendicular to the sialic acid residue and pointing out of the active site whilst in contrast, the angle made by the galactose to the sialic acid in the 6-SL structure is different and directs the galactose into the central cleft of the enzyme dimer. In both cases no hydrogen bonding network could be seen between galactose and the protein backbone, indicating it may exist in various orientations. The electron density for the third sugar of 3-SL (glucose) is not ordered and again suggests a degree of potential flexibility. This is the first example of a bacterial sialidase in complex with oligosaccharide sugars, and comparisons of this structure with the Influenza A N2 neuraminidase subunit correspond well with the position of 3-SL and 6-SL sugars and place key residues in very similar positions, suggesting a significant conservation across this family of GH34 (viral sialidases) and GH33 enzymes (Supplementary Figure S12).

### Structure–function studies of NanH

With the structural models of NanH in complex with one inhibitor and two native ligands, we set out on a programme of mutagenesis to probe several structure–function questions in relation to NanH and its dual-specificity and catalytic action. Firstly, while the role of the arginine triad is well established and present in our models, we wanted to probe several aspects of the role of the conserved residues in our enzyme, with a particular focus on the Tyr–Glu nucleophilic pair, which others have indicated may not be essential [48]. Specifically, Y518 along with E407 in *T. forsythia* NanH likely forms part of a proposed Tyr–Glu pair that acts to donate an electron during catalysis. Mutation of this residue to a range of amino acids to probe the role of charge and size resulted in inactive mutants and indicated that NanH Y518 is a key residue for activity (Figure 8), which is not always the case in the GH33 enzymes i.e. in *M. viridifaciens* [49] and *C. perfringens* [50], and suggests that NanH uses a typical retaining sialidase mechanism [16]. To confirm its role in catalysis we also mutated R212 (part of the arginine triad that co-ordinates the carboxylate group of sialic acid) which resulted in an inactive enzyme (Supplementary Figure 8B and Table S3).

Finally, while several studies have crystallised and constructed models of various bacterial and viral sialidases there is a dearth of information on the role of residues beyond the active site and their effects on catalysis or specificity for the glycosidic linkage. We targeted residues (N234, S235, V236) that were adjacent to key catalytic residues and that we predicted might constrain the substrate in 3D space since they were near the glycosidic linkage. Mutated variants were generated to test the effect of inserting or substituting large bulky residues that might affect substrate orientation but not inactivate catalysis. When comparing our structural model to that of other sialidases, we also noted that NanH lacked a Tyr–His pair that sits above the carboxylate group of sialic acid in several α2,3-specific sialidases (Y307 and H278 in *S. typhimurium*, Supplementary Figure S10); further analysis of other 2,3-specific enzymes also reveals bulky residues in this region (e.g. NanB *S. pneumoniae* and rgNanH from *R. gnavus*). We thus postulated that introducing bulky residues might constrain the substrate entrance/exit channel and influence specificity. To achieve this, we produced enzyme variants with a range of mutations in an attempt to mimic the H-Y pair as well as the bulky residues from other enzymes to assess how this influenced activity and specificity (e.g. S455+ H-G486 +Y, I456Y and N425W). As part of these studies, we also tested whether other mutations to D237 might be tolerated and even influence specificity, replacing D237 with glutamate, asparagine, glutamine and serine. Finally, using this idea that introducing bulky residues might constrain substrate mobility or entry/exit from the active site, we also introduced a mutation (A307Y) at the periphery of the active site. In addition, our enzyme and others present in *Bacteroidetes* [35] species contain an unusual rotamer for R306, which is part of a protruding loop present near the active site and which may also influence the position of the glycosidic bond of α2,3 or α2,6 specific substrates, and hence was targeted too. All mutated residues are illustrated on the catalytic domain structure (Figure 8A).

Figure 8B summarises the data obtained from these mutagenesis studies. Firstly all mutations to D237, resulted in fully soluble, but inactive enzymes (Figure 8B, Supplementary Table S3). In the case of D237E, this was unsurprising as even small changes to this key catalytic residue are not tolerated in GH enzymes. The mutations that introduced bulky residues (N425W, A307Y) in proximity to the active site or the H-Y pair (S455+H I456 G486+Y; + indicates to insertion of residues not occurring in natural enzyme) resulted in several inactive enzymes. In contrast other changes in the region close to the glycosidic bond (S235Y, I456Y and N234 ±Y S235) resulted in enzymes with generally reduced activity (i.e. reduced *k*_cat_*/k_M_*), but with an altered specificity (i.e. changed *k*_cat_*/K_M_* (α2,3): *k*_cat_*/K_M_* (α2,6) ratios). For example, while NanH-WT has a ratio of 2.2, for the *k*_cat_/*k_M_* values with the two substrates favouring turnover of the α2,3 substrate, the lower ratio values of the N234(+Y)S235 (1.7) and I456Y (1.6) enzymes suggest a shift in preference away from α2,3 towards α2,6 substrates when compared with the WT enzyme. Finally, the mutations to V236 (V236Y and V236Q, Supplementary Figure S11), in close proximity to the glycosidic linkage and D237 resulted in enzymes with ∼50% overall activity, but importantly specificity ratios of 1.2 and 1.45, respectively, and thus much closer to 1 : 1 than WT (2.2). Mutagenesis to alanine of R306 (Supplementary Figure S11) with the unusual arginine rotamer form also resulted in similar alterations in specificity (ratio of 1.3 with an activity of 54%). For all of these changes (except S235Y), the substrate preference seemed to reduce for α2,3 and increase for α2,6-linked sialic acid- indicating that these residues might be able to influence specificity, potentially by affecting binding or access of the different sugars based on the predicted position of the 3rd unresolved sugar and represent a target for future work, where combinations of mutations to these residues might yield new information. The only other study, to our knowledge, that successfully altered sialidase specificity was the mutation of a tryptophan (W312) that sits at the entrance to the active site of the transialidase from *T. cruzi*, where an α2,3 enzyme was altered to cleave both α2,3 and α2,6 linkages [51]. It is also notable that no solely α2,6 specific GH33 sialidase has yet been discovered to date [52], while several α2,3 specific enzymes are known [18]. The only enzyme capable of α2,6 specific desialylation activity reported to date is the α2,6-sialyltransferase from *Photobacterium* that will perform its transialidation reaction in reverse in the presence of an excess of CMP, and has been termed a pseudosialidase [52].

## Conclusions

The data presented here provide a structural understanding of a stable, efficient, broad-specificity enzyme with data highlighting how inhibitors as well as natural substrates interact with this key sialidase from *T. forsythia*. We also provide data for evaluating key residues in glycosidic linkage specificity that could pave the way for the directed evolution of NanH, to produce a solely α2,6 specific enzyme. Finally, the data provide the basis for both drug design research into selective inhibitors of this enzyme, to counteract the raised sialidase activity observed in periodontitis and ameliorate the disease, as well as providing insight into this key activity for the virulence of periodontal pathogens [3,4,24,46].

## Materials and methods

### Bacterial cell culture

*Escherichia coli* strains were cultured in 2xYT broth (Fisher Scientific) or on LB agar (Fisher Scientific) with antibiotics (Ampicillin, 50 μg ml^−1^) added as appropriate.

### Production of recombinant NanH and NanH point mutants

The *nanH* gene from *T. forsythia* 92A.2 was previously synthesised in an *E. coli* codon-optimised construct and sub-cloned into pET21a (+) (see [30] for more details). *E. coli* BL21(DE3) was transformed with expression plasmids and grown in 2xYT with 1 mM IPTG (isopropyl-β-d-thiogalactopyranoside) induction at OD_600 _= 0.6, for 18 h at 25°C with agitation. Cells were harvested and resuspended in 50 mM sodium phosphate, pH 7.4, 200 mM NaCl and 20 mM imidazole. For large-scale purification, cells were disrupted using a French pressure cell at 1050 psi (3X) (Thermo Scientific) and soluble fractions clarified by further centrifugation (15 000×***g***, 40 min, 4°C). The C-terminally hexahistidine-tagged proteins were purified using a 5-ml HisTrap FF Ni^2+^-sepharose affinity chromatography column (GE Healthcare) and eluted with a 0–500 mM imidazole gradient on an ÄKTAprime plus (GE Healthcare). Purified proteins were dialysed against 50 mM sodium phosphate, pH 7.4, 200 mM NaCl, concentrated using a MWCO 10 000 Vivaspin column (GE Healthcare) where necessary and their concentrations were determined using the Pierce BCA Protein Assay Kit (Thermo Scientific).

Alternatively, for testing of NanH site-directed mutagenesis (SDM) variants, purification was performed on a smaller scale with 50 ml cell pellets resuspended in buffer as above, disrupted using 3 × 3 s of sonication at 16 µm amplitude (30 s on ice between each pulse). This was followed by incubation with 200 µl equilibrated NiNTA nickel resin (Qiagen) loaded into Micro Bio-Spin chromatography columns (Bio-Rad), before washing with the same buffer and elution using 300 mM imidazole and dialysis as above.

### Site-directed mutagenesis

Point mutants of *nanH* were produced using the QuikChange II Site-Directed Mutagenesis Kit (Agilent Technologies) according to the manufacturer's instructions, transformed into *E. coli* XL-10-Gold and the sequences were verified using the Sanger sequencing by GATC Services (Eurofins Genomics); primer sequences available on request.

### Size-exclusion chromatography and size-exclusion chromatography-multiangle laser light scattering (SEC-MALLS)

Purified and dialysed WT NanH was concentrated using a Viva Spin (GE Healthcare Sciences) to ∼3 mg ml^−1^; 2 ml of concentrated NanH was loaded onto a 16 × 600 HiLoad Superdex column equilibrated in 0.5 M NaCl, 50 mM Tris, pH 8.0. The gel filtration involved the collection of 2 ml fractions at a flow rate of 1.5 ml min^−1^ using an AKTA Purifier 10 FPLC System. The fractions that formed the main peak on the chromatogram were combined and analysed by SDS–PAGE. The apparent molecular mass (MW) of the peak was determined by calculating the *K*_av_ and applying this to a standard curve of proteins of known MW's.

Purified WT NanH was further analysed by size-exclusion chromatography-multiangle laser light scattering (SEC-MALLS). NanH (0.5 and 3.0 mg ml^−1^) was analysed on a Superdex S200 10/300 column (GE Healthcare) in 50 mM sodium phosphate, 200 mM NaCl pH 7.4, at ∼20°C on a system comprising a Wyatt HELEOS-II multi-angle light scattering detector and a Wyatt rEX refractive index detector linked to a Shimadzu HPLC system (SPD-20A UV detector, LC20-AD isocratic pump system, DGU-20A3 degasser and SIL-20A autosampler). Data were analysed using the Astra V software (Wyatt Technologies). The experiment was performed by the Molecular Interactions Lab, University of York, U.K.

### Reaction kinetics of purified NanH and NanH variants with 4-methylumbelliferyl-*N*-acetyl-α-d-neuraminic acid

Reaction kinetics of NanH and NanH variants were obtained under the previously established pH optimum and under physiological pH 7 conditions [30]. Increasing concentrations of 4-methylumbelliferyl-*N*-acetyl-a-d-neuraminic acid (MU-NANA) (Carbosynth) were exposed to 2.5–100 nM of sialidase in 50 mM sodium phosphate, 200 mM NaCl, pH 7.4 and reactions quenched by addition of 100 mM sodium carbonate buffer, pH 10.5, every 1 min at a volume ratio of 1 : 1.5 (reaction : sodium carbonate). Sialidase activity was quantified by measuring 4-MU fluorescence (λ_ex _= 350 nm; λ_ex_^ ^= 450 nm) and application of a 4-MU standard curve to allow quantification of the 4-MU release rate. The rate of reaction was determined by first finding the initial rate of reaction, which occurred between 1 and 3 min for WT NanH and NanH variant. The initial rate, also referred to as velocity (*V*_0_, 4-MU release μmol min^−1^ mg^−1^), was plotted against MU-NANA concentration (μM) and the *K_M_* and *V*_max_ were determined in GraphPad PRISM 7.03 software by fitting the following nonlinear equation using the least-squares method:

$$Y=V_{\max}\times \frac{X}{K_{M}+X}$$

*X* refers to the substrate concentration, *Y* is the enzyme velocity, *V*_max_ is the maximal enzyme velocity and *K_M_* is the concentration at half-maximal velocity when the enzyme becomes saturated; assays were performed in triplicate for each variant. The *k*_cat_ values were determined in GraphPad PRISM 7.03 software by fitting the following nonlinear equation using the least-squares method:

$$Y=E_{t}\times k_{\mathrm{cat}}\times \frac{X}{K_{M}+X}$$

*X* refers to the substrate concentration, *Y* is the enzyme velocity, *E_t_* is the concentration of enzyme active sites, *k*_cat_ is the turnover number and *K_M_* the Michaelis–Menten constant.

### Reaction kinetics of NanH and NanH variants with host-relevant ligands

To obtain the glycosidic linkage preference of the NanH variants for α2,3- or α2,6-linked sialic acid (Neu5Ac), we used the thiobarbituric acid (TBA) acid assay adapted from Aminoff [53], as per our previous work [30]. Application of a Neu5Ac standard curve allowed quantification of free sialic acid. The initial rate was determined at all concentrations tested and Neu5Ac release (*V*_0_, Neu5Ac release µmol min^−1^ mg^−1^) was plotted against ligand concentration (mM) and the *K_M_, V*_max_ and *k*_cat_ were determined in GraphPad PRISM 7.03 software by fitting the nonlinear equations using the least-squares method.

### Sialidase inhibition assays

2.5 nM of purified WT NanH was incubated with 0.1 mM MU-NANA at room temperature in 50 mM sodium phosphate, 200 mM NaCl, pH 7.4, in the presence of increasing concentrations of inhibitor. Inhibitors studied in this project were *N*-acetyl-2,3-dehydro-2-deoxyneuraminic acid (DANA) (Carbosynth) and oseltamivir acid (Carbosynth). The reactions were incubated at room temperature as above with the addition of substrate to the reaction as the last step. Sialidase inhibition was expressed as the percentage change in fluorescence with a given concentration of inhibitor compared with fluorescence in the absence of inhibitor, as described by the formula below, where Top is 100% (activity), Bottom is the percent of activity at maximum inhibition and the IC_50_ is the inhibitor concentration which reduces activity by 50%.

$$Y=\frac{\text{Bottom}+(\mathrm{Top}-\text{Bottom})}{\left(1+\left(\frac{X}{IC_{50}}\right)\right)}$$

This equation was fitted to the data using GraphPad PRISM 7.03 [inhibitor] vs. response (three parameters) nonlinear fit (by least-squares method).

### Protein–ligand binding assay

The binding characteristics of the puriﬁed NanH–D237A variant with 3- and 6-SL was investigated by steady-state tryptophan ﬂuorescence spectroscopy (the variant possesses eleven tryptophan residues), effectively as in [30]. The protein was diluted to 0.1 µM in 50 mM sodium phosphate, 200 mM NaCl pH 7.4 and incubated with differing concentrations of ligand in the wells of an optically clear, ﬂat-bottom 96-well plate (Greiner) at 25^°^C for 5 min. The quenching of intrinsic tryptophan ﬂuorescence was measured at 25°C in a ﬂuorescence spectrometer (Tecan M200), with the excitation wavelength set to 280 nm (5 nm slit-width) and emission spectra recorded over a scan range of 300–380 nm (10 nm slit-width). The change in protein fluorescence due to an increase in the concentration of ligand was plotted as a percentage change relative to a protein only baseline condition. The *B*_max_ and *K_d_* of the ligands were determined using the following equation:

$$Y=B_{\max}\times \frac{X}{\left(K_{d}+X\right)}$$

*X* refers to the final concentration of ligand; *Y* is the change in protein fluorescence as a percentage compared with the protein only condition and *B*_max_ is the maximum number of binding sites. This equation was fitted to the data using GraphPad PRISM 7.03 one site-specific binding nonlinear fit (by least-squares method).

### Crystallisation

Crystallisation trials, using a variety of commercial screens (Molecular Dimensions) were performed on the purified WT NanH and NanH-D237A (20–40 mg ml^−1^) after dialysis of protein into 10 mM Tris, 100 mM sodium chloride (NaCl), pH 7.5. Initial screening for the WT was performed using a Matrix Hydra II PlusOne robot (Thermo Scientific), which dispensed into 96-well sitting-drop trays (Molecular Dimensions), where a 1 : 1 ratio of precipitant: protein generated 400 nl drops, that were allowed to equilibrate with the crystallization reagent reservoir solution by vapour diffusion at 290 K. Successful crystallisation conditions for each construct can be found in Table 1. Hits from the initial screens were optimised using the hanging drop technique in 24-well plates with a reservoir volume of 500 µl and a drop size of 4 µl. A Mosquito (TTP Labtech) was used for the crystallisation trials of the NanH-D237A variant, using a 1 : 1 ratio of precipitant: protein and 200 nl drops.

### Data collection, crystal soaking and processing

Crystals were harvested using cryoloops (Hampton Research) and immediately soaked in a cryoprotectant solution containing the components of the crystallisation reservoir solution, which was supplemented with 15% ethylene glycol. In some cases, 5 mM inhibitor or ligand was added for soaking of crystals — before being flash cooled in liquid nitrogen. Crystals produced in this project were subjected to X-ray radiation at the Diamond Synchrotron Light Source near Oxford (U.K.) on I02, I03, I04 and I24 beamlines. Diffraction data were collected at 100K shown in Table 1 and the data were indexed and integrated to produce a complete dataset using the xia2 XDS software [54] at the Diamond Light Source. The AIMLESS program from the CCP4 suite of programs [55] was used to merge the symmetry-related reflections to generate a set of unique diffraction intensity data and to assess the data quality. Calculated values for the space group, mosaicity and unit cell dimensions of the crystal were obtained.

### Structure determination and refinement

The CCP4 suite of programs [55] was used to manipulate the data and enabled the calculation of the Matthews coefficient (*V_m_*) [56] to estimate the number of protein molecules in the asymmetric unit. Molecular replacement was used to solve the crystal structure of native NanH using the PHASER program from the CCP4 suite [55,57] with the BTSA sialidase from *B. thetaiotaomicron* as the search model (PDB: 4BBW). The models underwent several rounds of model building in Coot (CCP4 suite) [58] and refinement in Refmac5 [55,59] and are deposited in the PDB (7QYP, 7QZ3, 7QY9, 7QYJ, 7QY8 and 7QXO).

### Analysis of procainamide labelled glycans

FA2G2S2 (A2F) glycan (Ludger Ltd.) underwent procainamide labelling (see below), as were glycans released from human plasma (Sigma) and recombinant human EPO as previously described [24]. Briefly, human EPO was expressed in Chinese hamster ovary (CHO) cells (a gift from Antonio Vallin, Center for Molecular Immunology, La Habana, Cuba). N-glycans were released using peptide N-glycosidase F (PNGase F, E-PNG01; Ludger Ltd.). EPO (in 17.5 µl) and plasma (10 µl diluted with 7.5 µl of pure water) were denatured at 100°C for 5 min with the addition of 6.25 µl 2% (w/v) SDS, 1 M 2-mercaptoethanol, then incubated at 37°C for 16 h with 1 µl PNGase F and 1.25 µl 15% (w/v) Triton X-100. Released N-glycans were fluorescently labelled with procainamide as described previously [24] using a LT-KPROC-96 kit (Ludger Ltd.). The released glycans were incubated with labelling reagents for 1 h at 65°C. The procainamide labelled glycans were cleaned up using LC-PROC-96 clean-up plate (Ludger Ltd.), then incubated with 1 μl of 1 mg ml^−1^ NanH in a final volume of 10 μl (50 mM sodium acetate buffer, pH 5.5) for 16 h at 37°C. Glycans were cleaned up again and NanH removed using a LC-PBM-96 clean-up plate (Ludger Ltd.). Procainamide labelled glycans (Ludger) were then analysed by UHPLC-FLD-ESI-MS/MS. Here, 25 µl of each sample was injected into a Waters ACQUITY UPLC Glycan BEH Amide column (2.1 × 150 mm, 1.7 µm particle size, 130 Å pore size) at 40°C on a Dionex Ultimate 3000 UHPLC instrument with a fluorescence detector (*λ*_ex_ = 310 nm, *λ*_em_ = 370 nm) attached to a Bruker Amazon Speed ETD. Mobile phase A was a 50 mM ammonium formate solution (pH 4.4) and mobile phase B was neat acetonitrile. Analyte separation was accomplished by a gradient running from 76% to 51% mobile phase B over 70 min at a flow rate of 0.4 ml min^−1^. The Amazon Speed was operated in the positive sensitivity mode using the following settings: source temperature, 180 C; gas flow, 4 l min^−1^; capillary voltage, 4500 V; ICC target, 200 000; maximum accumulation time, 50.00 ms; rolling average, 2; number of precursor ions selected, 3; scan mode, enhanced resolution; mass range scanned, 400–1700. Data were analysed using Bruker Compass Data Analysis software v4.1 and glycan diagrams made using GlycoWorkbench v2.0. Glycan compositions were elucidated based on MS2 fragmentation.

## Data Availability

All structural data in this manuscript are deposited in the PDB under the following accession numbers: 7QYP [60], 7QZ3 [61], 7QY9 [62], 7QYJ [63], 7QY8 [64], 7QXO [65].

## Abbreviations

CBM: carbohydrate-binding modules
EIC: extracted ion chromatogram
EPO: erythropoietin
FT: freeze–thaw
MW: molecular mass
TBA: thiobarbituric acid
