# Supplementary material for: Structural and functional characterisation of a stable, broad-specificity multimeric sialidase from the oral pathogen Tannerella forsythia
Source: Biochem J. 2022 Sep 6;479(17):1785–806. doi: 10.1042/BCJ20220244 (PMC9472817; doi:10.1042/BCJ20220244)
Supplement: Supplementary Material [file BCJ-479-1785-s1.pdf]

SUPPLEMENTARY INFORMATION

Table S1- Identity of EPO undigested N-glycans. FILE: TABLE S1

Table S2- The RMS on the xyz displacement of the Cα's for each of the 519 residues when NanH-D237A subunits were superimposed onto the equivalent subunit of NanH-HEPES. SUPERPOSE program from the CCP4 software suite was used to superimpose subunits A and B from the NanH-D237A models onto the equivalent subunits in the NanH-HEPES model.

**Fixed**	**Moving**	**RMS (Å)**
NanH-HEPES CA	D237A-apo CA	0.15
NanH-HEPES CB	D237A-apo CB	0.17
NanH-HEPES CA	D237A-3SL CA	0.15
NanH-HEPES CB	D237A-3SL CB	0.15
NanH-HEPES CA	D237A-6SL CA	0.14
NanH-HEPES CB	D237A-6SL CB	0.15

Table S3 Kinetic parameters for NanH and all mutant variants as indicated with MU-NANA substrate.

**Variant**	**V_{max} (µmol min^{-1} mg^{-1})**	**K_M (µM)**	**k_{cat} (min^{-1})**	**k_{cat}/K_M (min^{-1} µM^{-1})**
**NanH-WT**	**13.8 ± 0.2**	**6.2 ± 0.5**	**799.6 ± 13.7**	**129.0**
**N234 +Y S235**	**10.6 ± 0.2**	**16.6 ± 1.2**	**613.8 ± 11.2**	**37.0**
**S235Y**	**7.1 ± 0.2**	**7.2 ± 0.9**	**412.7 ± 10.5**	**57.3**
**V236Q**	**2.4 ± 0.1**	**2.5 ± 0.6**	**140.0 ± 5.4**	**56.0**
**V236Y**	**4.8 ± 0.1**	**4.4 ± 0.6**	**278.6 ± 7.7**	**63.3**
**D237A/E/Q/S/N**	**N/A**	**N/A**	**N/A**	**N/A**
**R306A**	**4.8 ± 0.1**	**4.0 ± 0.4**	**279.9 ± 4.8**	**70.0**
**A307Y**	**N/A**	**N/A**	**N/A**	**N/A**
**N425W**	**N/A**	**N/A**	**N/A**	**N/A**
**S455 +H**	**N/A**	**N/A**	**N/A**	**N/A**
**I456Y**	**5.7 ± 0.1**	**18.8 ± 1.3**	**328.5 ± 6.0**	**17.5**
**G486 +Y**	**N/A**	**N/A**	**N/A**	**N/A**
**Y518A/Q/S/T/F**	**N/A**	**N/A**	**N/A**	**N/A**

Table S4 Kinetic parameters of NanH and variants with 3-SL or 6-SL as assayed by TBA assay. In both cases reaction kinetics values were obtained from Michaelis-Menten curves of NanH variants with the indicated substrate. The V_{max}, K_M, k_{cat} and k_{cat}/K_M (efficiency) values are presented (± standard error). N/A indicates Non-active**.**

	3-sialyllactose				6-sialyllactose			
**Variant**	V_{max} (µmol min^{-1} mg^{-1})	K_M (mM)	k_{cat} (min^{-1})	k_{cat}/K_M (min^{-1} mM^{-1})	V_{max} (µmol min^{-1} mg^{-1})	K_M (mM)	k_{cat} (min^{-1})	k_{cat}/K_M (min^{-1} mM^{-1})
**NanH-WT**	46.4 ± 1.2	0.3 ± 0.0	2683.0 ± 69.7	8654.8	43.4 ± 1.1	0.6 ± 0.1	2509.0 ± 63.5	3920.3
**S235Y**	37.2 ± 5.3	0.5 ± 0.2	2153.0 ± 305.2	4306	14.5 ± 1.5	0.5 ± 0.2	836.1 ± 86.3	1672.2
**N234 +Y S235**	15.5 ± 1.8	0.4 ± 0.2	899.6 ± 103.3	2249	32.4 ± 4.7	1.4 ± 0.5	1877.0 ± 269.6	1340.7
**V236Q**	33.1 ± 0.7	0.3 ± 0.0	1910.0 ± 43.3	6366.7	30.4 ± 0.8	0.4 ± 0.0	1759.0 ± 44.4	4397.5
**V236Y**	48.8 ± 1.2	0.5 ± 0.0	2828.0 ± 69.6	5656	41.1 ± 1.4	0.5 ± 0.1	2373.0 ± 80.3	4746
**D237A/E/Q/S/N**	N/A	N/A	N/A	N/A	N/A	N/A	N/A	N/A
**R306A**	47.4 ± 1.7	0.5 ± 0.1	2738.0 ± 99.8	5476	43.5 ± 1.3	0.6 ± 0.1	2510.0 ± 72.9	4183.3
**A307Y**	N/A	N/A	N/A	N/A	N/A	N/A	N/A	N/A
**N425W**	N/A	N/A	N/A	N/A	N/A	N/A	N/A	N/A
**S455 +H**	N/A	N/A	N/A	N/A	N/A	N/A	N/A	N/A
**I456Y**	5.9 ± 1.1	0.6 ± 0.4	340.7 ± 65.2	567.8	6.0 ± 0.8	1.0 ± 0.4	348.9 ± 44.1	348.9
**G486 +Y**	N/A	N/A	N/A	N/A	N/A	N/A	N/A	N/A
**Y518A/Q/S/T/F**	N/A	N/A	N/A	N/A	N/A	N/A	N/A	N/A

Fig. S1

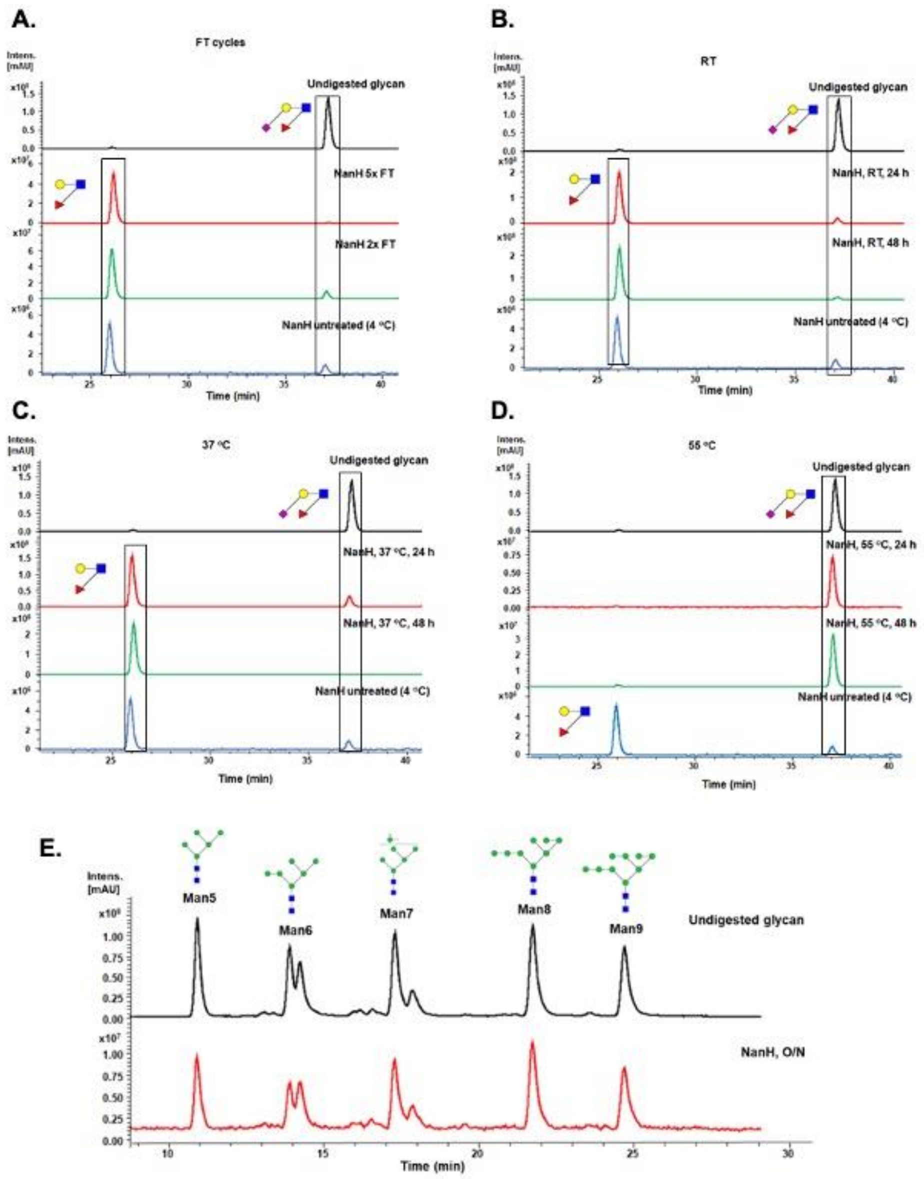

Figure S1: LC-FLR of procainamide labelled NanH digest of 3-Sialyl-Lewis X (SLe^{X}) when NanH was subjected to different conditions. NanH digest (O/N 0.1 mgml^{-1}) of SLe^{X} after NanH was subjected to various conditions, in all panels the black chomatogram represents undigested SLe^{X}.: (A) Five (5x- Red trace) or two (2x- Green trace) Freeze Thaw cycles (FT) or stored at 4°C (blue trace). (B) Undigested glycan (black trace) is compared with NanH stored at Room Temp (20C) for 24 (red) or 48h (green) or 4°C (blue trace). (C/D) Undigested glycan (black trace) is compared with NanH stored at 37C or 55C for 24 (red) or 48h (green) as shown. **(E) LC-FLR of procainamide labelled NanH digest of MAN Mix.** NanH digest (O/N 0.1 mgml^{-1}) of MAN Mix (Ludger) after NanH was subjected to various conditions. Undigested

MAN Mix is represented by the black trace with each peak represented by the appropriate MAN glycan. The red trace represents the results of the MAN Mix NanH digest with the peaks being identical to that of the undigested samples and therefore the same glycans as shown on the undigested trace. NOTE: Peaks are labelled according to standard annotation.

Fig. S2

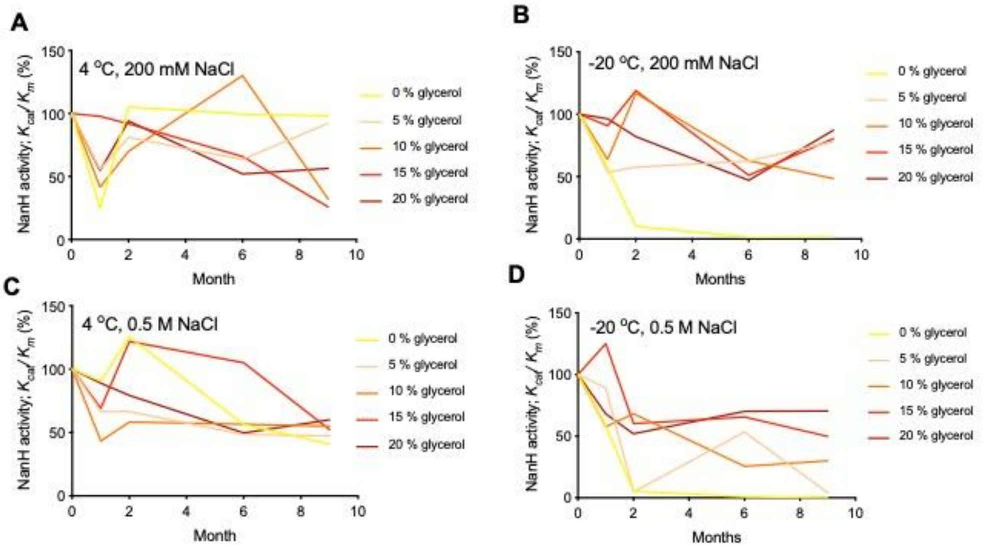

E

	37 °C, 24 h	37 °C, 48 h	42 °C, 24 h	42 °C, 48 h	4 °C
V_{max} (µmol min^{-1} mg^{-1})	4.4 ± 0.1 (4.3 to 4.6)	6.9 ± 0.3 (6.4 to 7.5)	0.8 ± 0.1 (0.7 to 1.0)	1.1 ± 0.2 (0.8 to 2.3)	6.0 ± 0.1 (5.7 to 6.3)
K_M ([MU-NANA], µM)	4.2 ± 0.5 (3.2 to 5.4)	5.8 ± 1.3 (3.4 to 8.8)	8.1 ± 3.2 (2.8 to 17.7)	110.1 ± 49.2 (44.7 to 395.1)	4.6 ± 0.7 (3.1 to 6.3)
k_{cat} (min^{-1})	255.3 ± 4.8 (245.7 to 265.2)	398.6 ± 15.4 (368.1 to 430.6)	47.8 ± 3.8 (40.4 to 56.3)	64.8 ± 13.0 (45.4 to 131.4)	344.6 ± 8.3 (326.5 to 363.2)
k_{cat}/K_M (min^{-1} µM^{-1})	60.9	68.8	5.9	0.6	75.7

Figure S2: Storage assay data shown as NanH % activity of *Kcat/ Km*. (A-D) MU-NANA reaction kinetics was performed for each individual storage condition at 1, 2, 3, 6- and 9-months post purification. Pure NanH was dialysed to 50 mM sodium phosphate, 200 mM NaCl, pH 7.4 and stored in 20µl aliquots under the conditions shown at 1 µM. The K_{cat}/K_M was calculated in assays using 2.5 nM enzyme to derive kinetics as before with MU-NANA substrate. Data were plotted as a percentage when the 0-month value (set as 100%) (A) Data for aliquots stored at 4 °C, 200 mM NaCl (B) -20 °C, 200 mM NaCl, (C) 4 °C, 0.5M NaCl and (D) -20 °C, 0.5 M NaCl; for up to 9 months. **(E) Accelerated degradation.** Table shows Michaelis-Menten kinetic parameters for MU-NANA when NanH (same purification as above) after being incubated for temperatures and duration shown. The V_{max}, K_M, K_{cat} and k_{cat}/K_M values are presented along with the associated error (standard deviation) for the reaction kinetics of MU-NANA and NanH.

Fig. S3

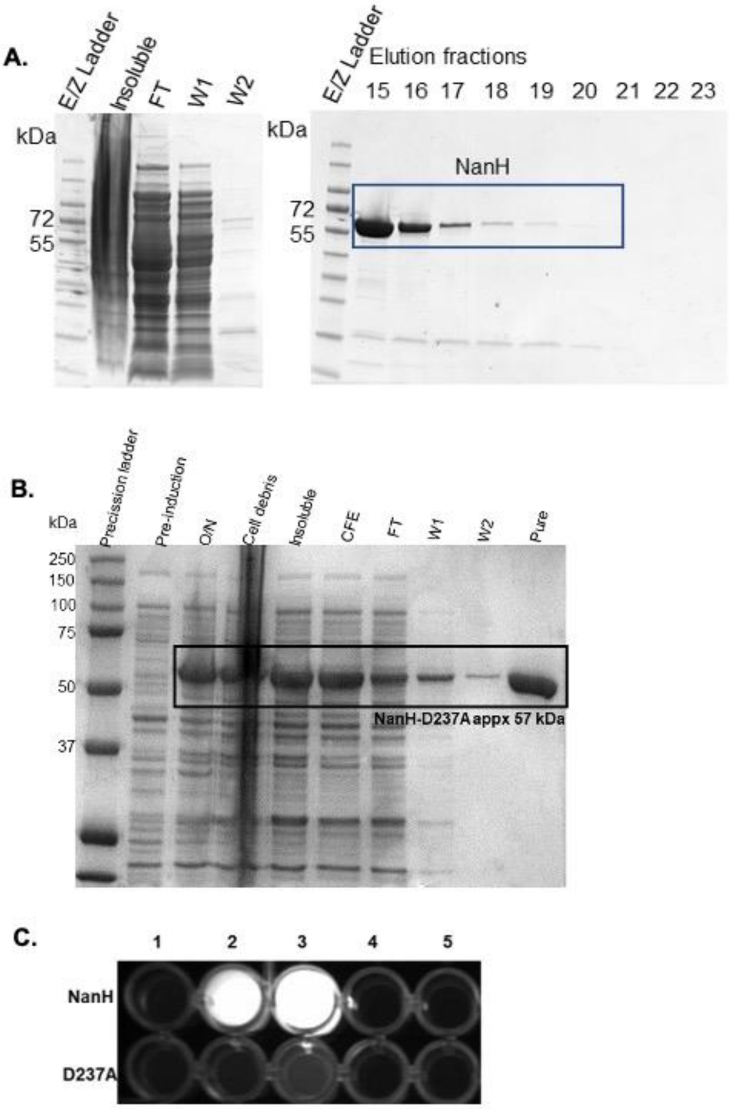

Figure S3: SDS-PAGE of NanH-WT (A) and D237A (B) by Ni-NTA purification (see methods). FT (Flow Through), W (wash); CFE (Cell Free soluble Extract). (C) Activity check using Mu-NANA (0.1mM) and visualised under ultra-violet light with (1) 0.1mM Mu-NANA control; (2) 10nM enzyme plus substrate); (3) 100nM enzyme plus substrate; (4,5) 10, 100nM enzyme alone.

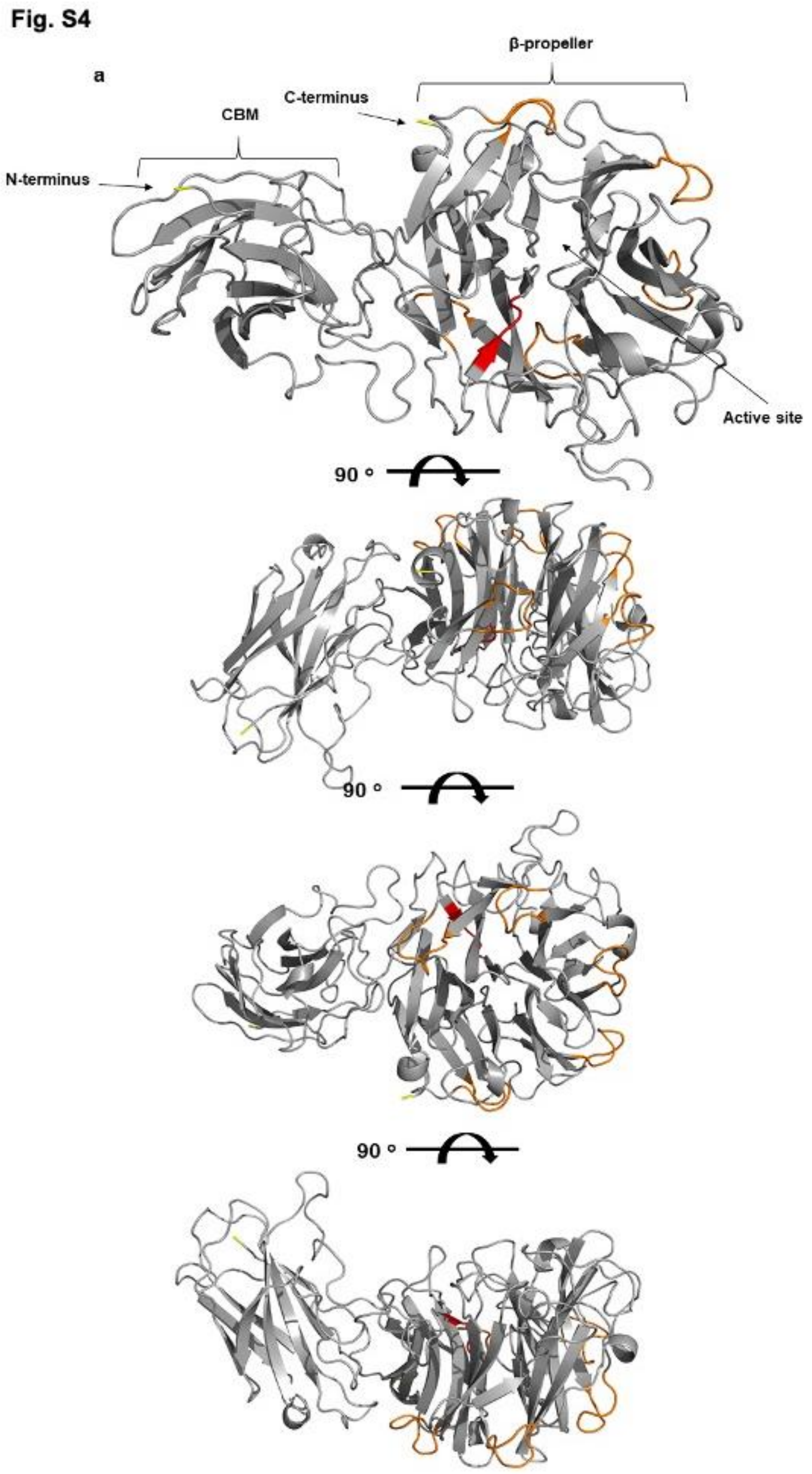

Figure S4: The crystal structure of a NanH-apo subunit with the FRIP motif (red) and Asp-boxes (orange) highlighted, presented in 4 views rotated around the horizontal axis by 90°. The N and C-termini are highlighted in yellow and labelled.

Fig. S5

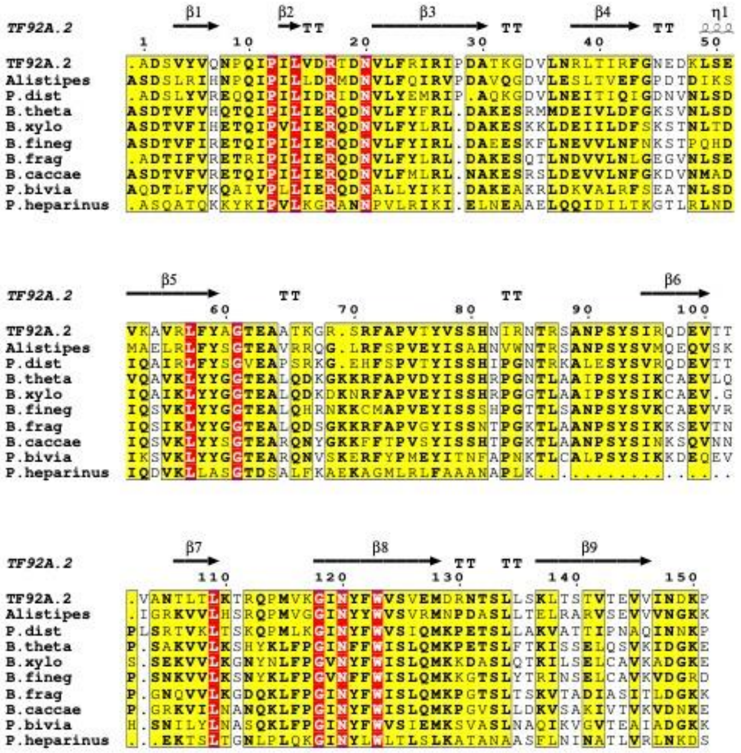

Figure S5: Alignment of NanH-CBM with other PF14873 family members. The alignment and image production were performed using MultAlin and ESPript using the NanH-apo pdb as a basis for secondary structure alignments. Strictly conserved residues are shown in white with a red background. The alignment consists of CBMs from the sialidases from *T. forsythia* (TF) strain 92A2 (WP_046826229.1), Alistipes sp., NCBI Taxonomy ID: 1262695 (CDD16645.1), *P. distasonis (*WP_011967072.1), *B. finegoldii (*CDC53424.1)*, B. thetaiotaomicron (*WP_008766031.1)*,* one of the *B. fragilis* sialidases (strain ATCC 25285, nanH1)(WP_008766031.1), *B. caccae (*CCZ75115.1)*, B. xylanisolvens (*CBK66099.1)*,* Prevotella bivia (WP_004336683.1) *Pedobacter. heparinus (*WP_015808031.1).

Fig. S6

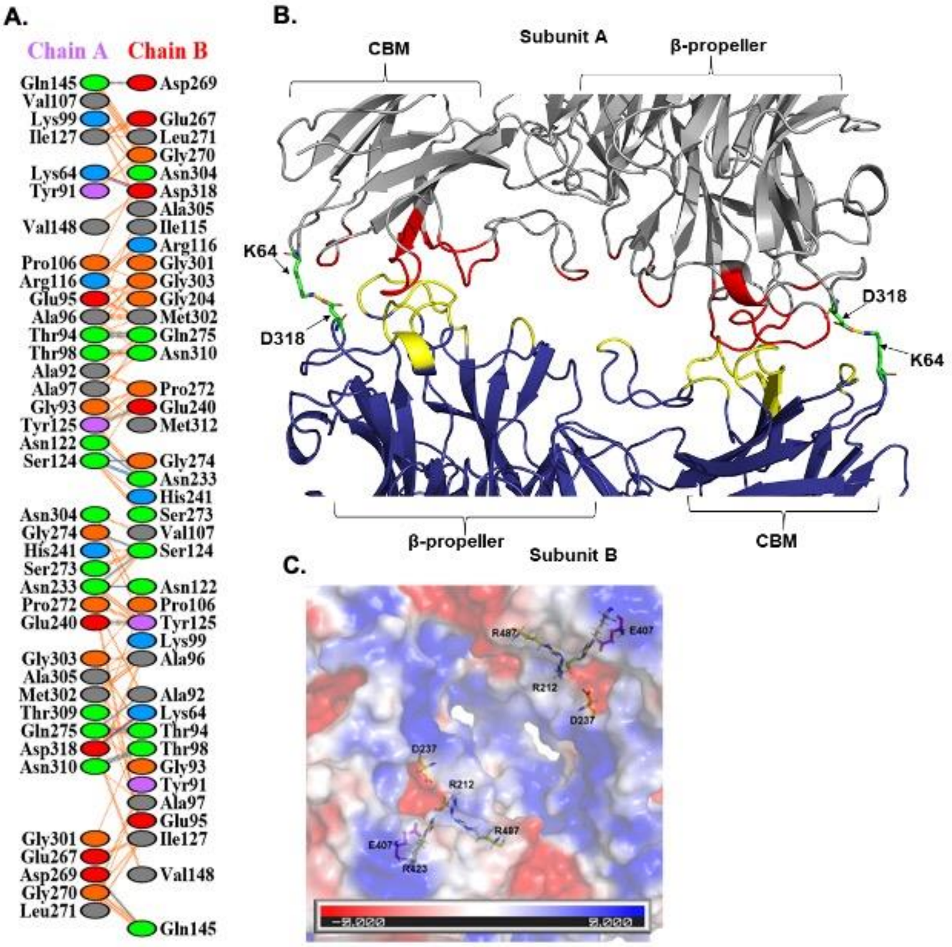

Figure S6: The protein-protein interface between subunit A and subunit B of NanH.

(A) A schematic diagram of the interactions between subunit A and subunit B. Salt bridges are represented by red lines, non-bonded contacts by orange dashed lines and hydrogen bonds by blue lines. Positively charged residues are shown in blue, negatively charged in red, neutral in green, aliphatics in grey, aromatics in purple, proline and glycine in orange and cysteine in yellow. The figure was created using PDB sum from EMBL-EBI.

(B) The protein-protein interface residues determined by PDBePISA are highlighted on the dimeric structure of NanH. The interface residues from subunit A (grey) are highlighted in red and those from subunit B (blue) are highlighted in yellow. The residues involved in salt bridge formation are highlighted as green sticks and labelled.

(C). Charge density surface map of the active site cleft in NanH.

Figure S7

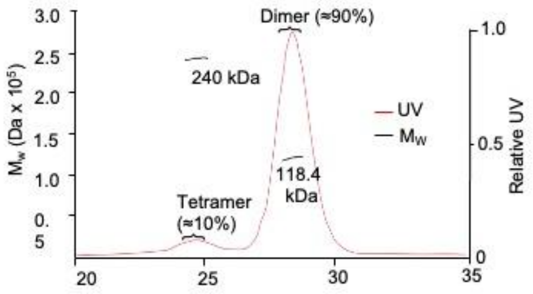

Figure S7. SEC-MALS trace of purified NanH showing multimeric forms of NanH corresponding to dimer and tetramer.

Fig. S8

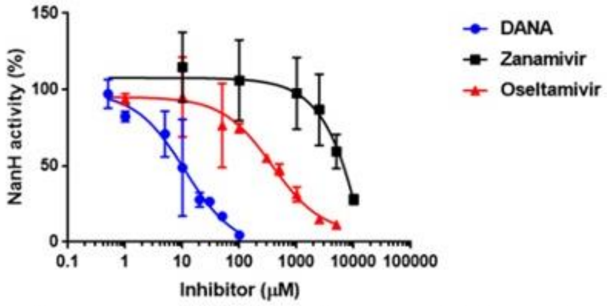

Inhibitor	Inhibitory constant (IC50) - NanH
N-Acetyl-2,3-dehydro-2-deoxyneuraminic acid (DANA)	11.0 ± 3.5 μM
Oseltamivir acid	371.6 ± 114.1 μM
Zanamivir	6309.0 ± 8.3 μM

Figure S8: The effect of inhibitors on NanH activity. The [inhibitor] was plotted (on a logarithmic, base 10, scale) against the percentage of sialidase activity relative to a no inhibitor control. The data represents the mean of three repeats and the error bars represent standard deviation. The IC_{50} values (lower panel-table) were calculated from the curves (± standard error).

Fig. S9

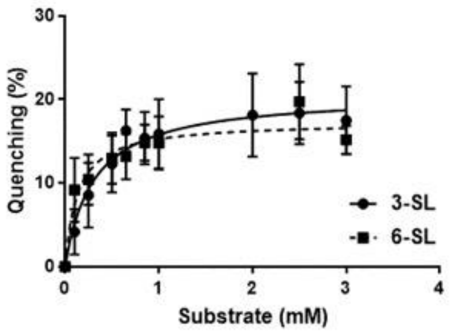

Substrate	Binding affinity (K_d) (μM)
3-SL	304.8 ± 91.5
6-SL	133.5 ± 48.3

Figure S9: NanH-D237A ligand binding assay. 0.1 μM NanH-D237A was exposed to increasing concentrations of 3-/6-SL and the change in protein fluorescence (%) compared to a no ligand dataset was used to obtain the binding affinity (K_d) of 3-/6-SL with NanH-D237A. The data presented represents the mean of 3 repeats, with the error bars representing standard deviation. **(Lower panel)** The K_d values are presented (± standard error).

Fig. S10

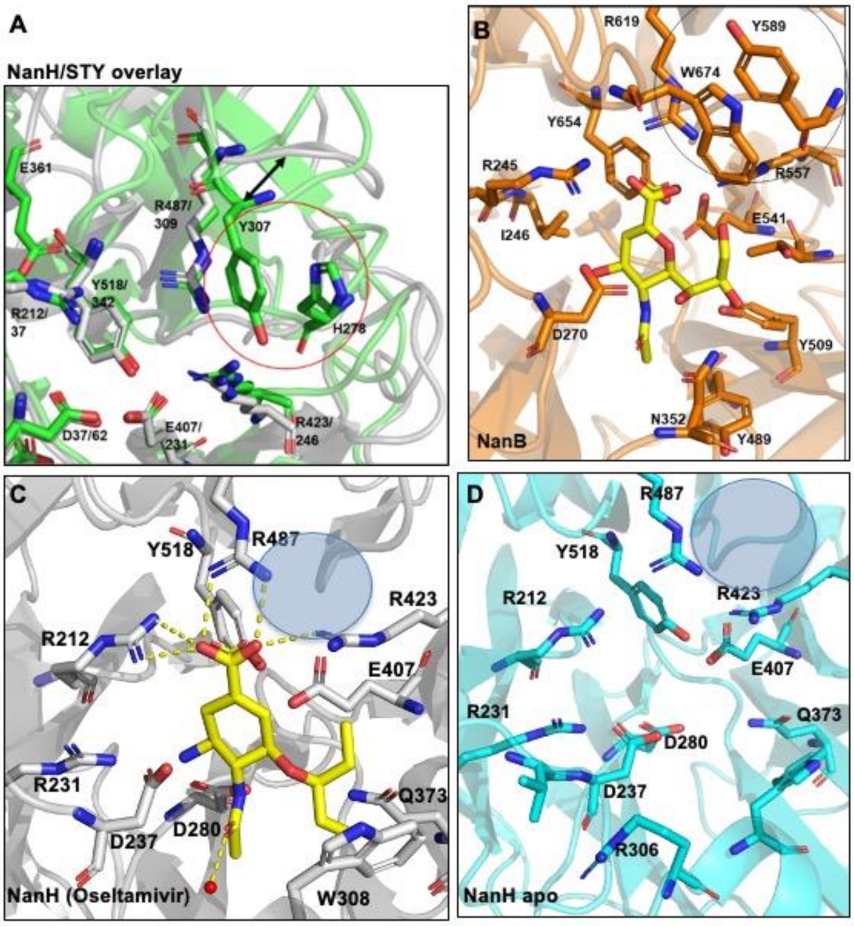

Figure S10: Location of H-Y pair and H-W pair in 2,3 specific sialidases in relation to NanH.

(A) Active site of sialidase from S. typhimurium (STY) (green residues) overlaid on NanH structure (grey residues). The circle indicates the Y-H pair, while an arrow indicates the large loop found in the *S. typhimurium* model.

(B) Active site of NanB from S. pneumoniae(PDB: 2VW1) in complex with DANA, circle highlights Y-W stack above the COOH co-ordination site**.**

(C)/(D) NanH in complex with Oseltamivir (C) and in apo form (D), highlighting the region where bulky residues are found in other related structures.

Figure S11

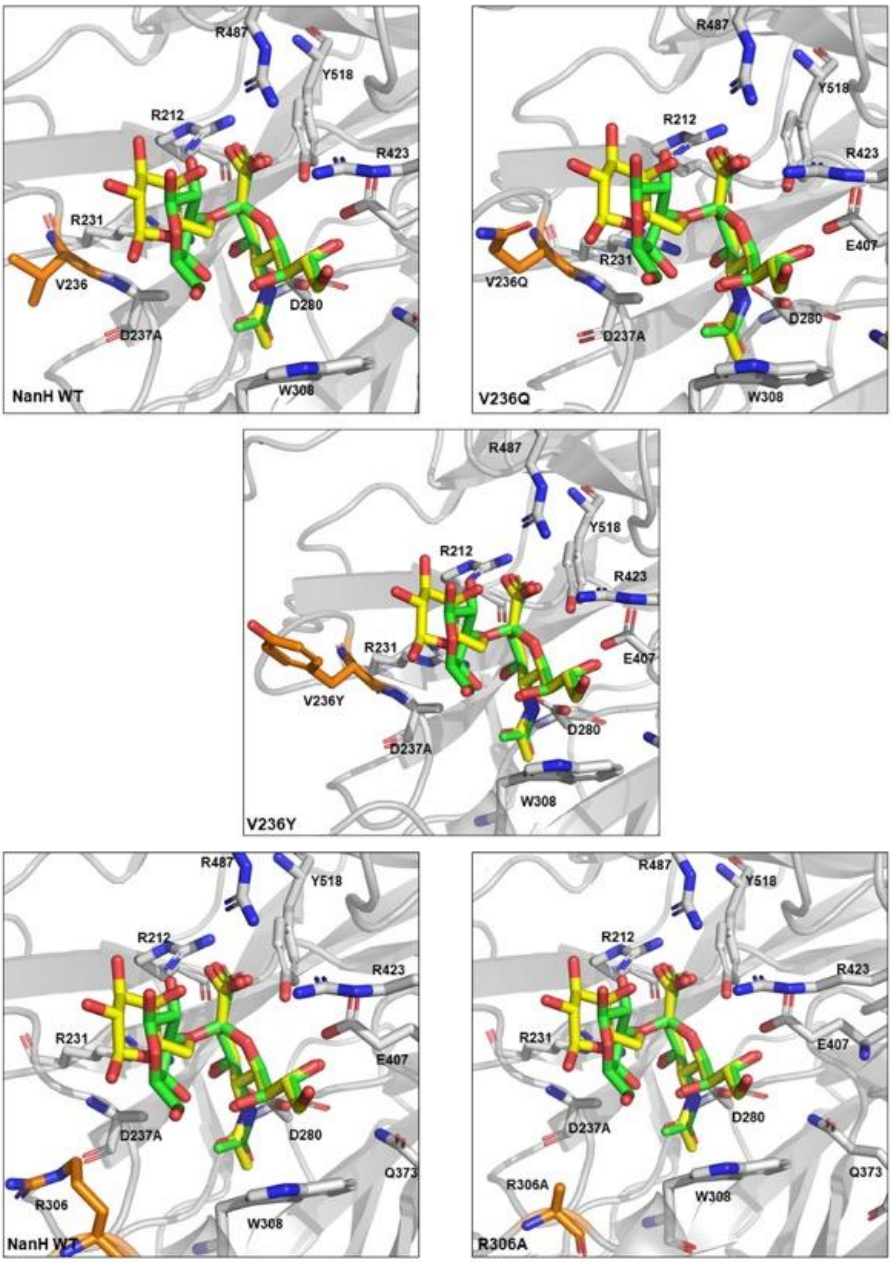

Figure S11: Location and predicted structure of mutations to V236 and R306. All figures show the active site with the key conserved residues and the surrounding region of NanH-D237A in complex with 3- and 6-SL (green and yellow sticks respectively, overlaid) and mutated target residue in orange. Figures were generated using the 'Mutagenesis Wizard' in PyMOL.

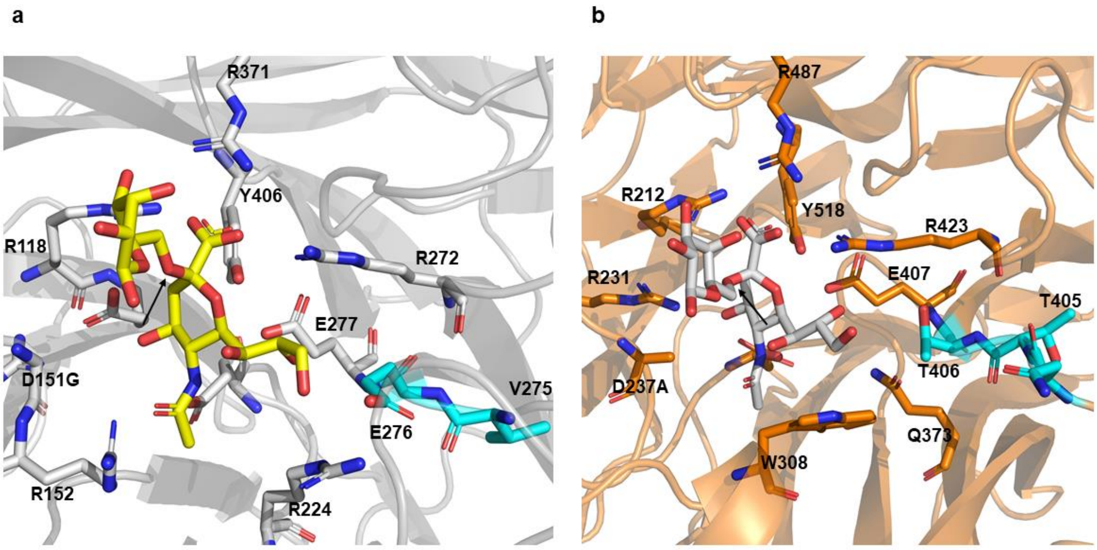

Figure S12: Comparison of active site 6-SL bound Influenza N2 and NanH from *T. forsythia*
The active site of N2 NA (grey) (PDB: 4GZX) in complex with 6'-SLN (yellow sticks). D151G is equivalent to D237A. (b) The active site of *T. forsythia* NanH (orange) in complex with 6-SL (grey sticks). In both images, the glycosidic linkage in each substrate is indicated by a black arrow.

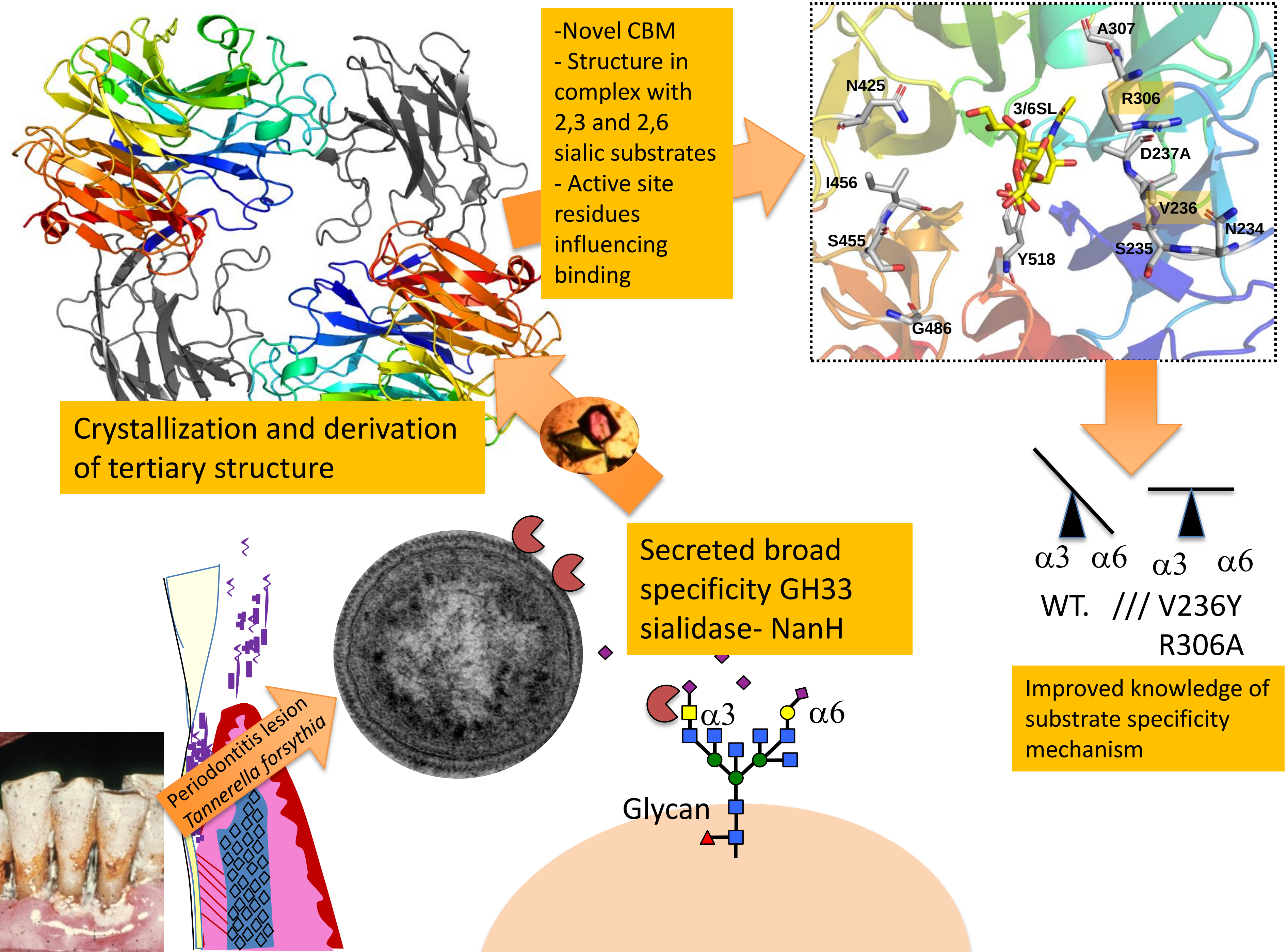
-Novel CBM
- Structure in complex with 2,3 and 2,6 sialic substrates
- Active site residues influencing binding
A307
N425
R306
3/6SL
D237A
I456
V236
N234
S455
S235
Y518
G486
Crystallization and derivation of tertiary structure
Secreted broad specificity GH33 sialidase- NanH
α3 α6
α3 α6
WT. /// V236Y
R306A
Improved knowledge of substrate specificity mechanism
α3
α6
Glycan
Periodontitis lesion
Tannerella forsythia

UHPLC Peak ID	% Area	ESI-LC/MS																		
		Composition									H+ adducts									
		Possible structure	Example Glycan cartoon	Hex (H)	HexNAc (N)	Fuc (F)	Neu5Ac (S) 0 OAc	Neu5Ac (S) 1 OAc	Neu5Ac (S) 2 OAc	potential sulphate or phosphate	$[M/Z]^{+}$ calculated	$[M/Z]^{+}$ observed	$[M/Z]^{2+}$ calculated	$[M/Z]^{2+}$ observed	$[M/Z]^{3+}$ calculated	$[M/Z]^{3+}$ observed	$[M/Z]^{4+}$ calculated	$[M/Z]^{4+}$ observed	$[M/Z]^{5+}$ calculated	$[M/Z]^{5+}$ observed
1	0.16	Man4+P	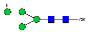	4	2	0	0	0	0	1	1372.52	1372.59	686.77	686.90	458.18	nd	-	-	-	-
2	0.45	FMan4+P	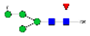	4	2	1	0	0	0	1	1518.58	1518.66	759.79	759.72	506.86	nd	-	-	-	-
3	1.23	Man5+P	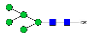	5	2	0	0	0	0	1	1534.57	1534.67	767.79	767.90	512.19	512.20	-	-	-	-
4	0.15	Man5+P	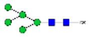	5	2	0	0	0	0	1	1534.57	1534.67	767.79	767.39	512.19	512.48	-	-	-	-
		FA2G2S2(Ac)2	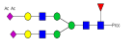	5	4	1	1	0	1	0	-	-	1337.02	1336.55	891.69	891.51	-	-	-	-
5	0.70	Man5+P	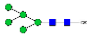	5	2	0	0	0	0	1	1534.57	nd	767.79	767.90	512.19	nd	-	-	-	-
		FMan5+P	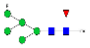	5	2	1	0	0	0	1	1680.64	nd	840.82	840.93	560.88	nd	-	-	-	-
6	0.47	FMan5+P	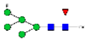	5	2	1	0	0	0	1	1680.64	nd	840.82	840.45	560.88	nd	-	-	-	-
		FA2G2S2(Ac)1	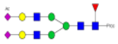	5	4	1	1	1	0	0	-	-	1316.02	1316.08	877.68	877.12	-	-	-	-
7	1.67	Man6+P	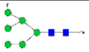	6	2	0	0	0	0	1	1696.63	1696.68	848.81	848.95	566.21	566.32	-	-	-	-
8,9	1.16	FA2G2S1	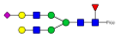	5	4	1	0	0	0	0	575.24	nd	1149.47	1149.54	766.65	766.76	-	-		-
		Man6+P	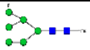	6	2	0	0	0	0	1	1696.63	1696.75	848.81	848.89	566.21	566.33	-	-	-	-
10,11	0.62	FMan6+P	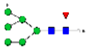	6	2	1	0	0	0	1	1842.69	nd	921.85	921.95	614.90	614.46	-	-	-	-
		Man6+P	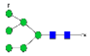	6	2	0	0	0	0	1	1696.63	nd	848.81	848.91	566.21	565.95	-	-	-	-
12	3.05	FA2G2S2	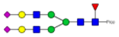	5	4	1	2	0	0	0	-	-	1295.01	1294.53	863.68	863.18	648.01	647.94	-	-
13	0.48	FA2G2S1S1(Ac)2	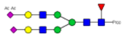	5	4	1	1	0	1	0	-	-	1337.02	nd	891.69	890.38	-	-	-	-
		FA3G3S2(Ac)1	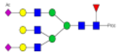	6	5	1	1	1	0	0	-	-	1498.56	nd	999.39	999.45	749.80	749.84	-	-
		FA2G2S2	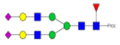	5	4	1	2	0	0	0	-	-	1295.01	1294.07	863.68	863.12	648.01	nd	-	-
			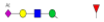																	

		FA3G3S3(Ac)2	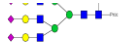	6	5	1	3	0	1	0	-	-	1665.14	nd	1110.43	1109.72	833.07	832.67	-	-
14,15	0.46	FA2G2S2	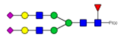	5	4	1	2	0	0	0	-	-	1295.01	1295.06	863.68	863.77	648.01	nd	-	-
16	0.94	FA3G3S1	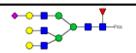	6	5	1	1	0	0	0	-	-	1332.03	1332.08	888.36	887.77	-	-	-	-
17	0.54	FA3G3S1	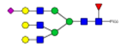	6	5	1	1	0	0	0	-	-	1332.03	1332.04	888.36	888.12	-	-	-	-
		FA4G4S3(Ac)2.	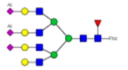	7	6	1	1	2	0	0	-	-	-	-	1232.14	1232.15	924.36	923.74	-	-
18	1.96	FA3G3S2	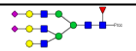	6	5	1	2	0	0	0	-	-	1477.58	1476.56	985.39	984.83	739.29	738.91	-	-
		A3G3S2(Ac)4	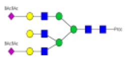	6	5	0	0	0	2	0	-	-	-	-	992.72	992.45	744.79	744.90	-	-
19	1.62	FA3G3S2	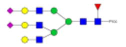	6	5	1	2	0	0	0	-	-	1477.58	1477.00	985.39	984.79	739.29	738.90	-	-
		A3G3S2(Ac)4	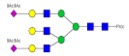	6	5	0	0	0	2	0	-	-	-	-	992.72	992.47	744.79	744.41	-	-
		FA4G4S3(Ac)2	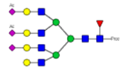	7	6	1	1	2	0	0	-	-	-	-	1232.14	1231.75	924.36	924.47	-	-
		FA4G4S4(Ac)2	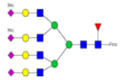	7	6	1	2	2	0	0	-	-	-	-	1329.17	nd	997.13	997.22	-	-
20	5.32	FA3G3S3	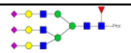	6	5	1	3	0	0	0	-	-	1623.13	nd	1082.42	1081.87	812.07	812.48	-	-
21	1.76	FA4G4S1 or FA3G3S1(LacNAc)1	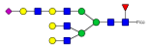	7	6	1	1	0	0	0	-	-	1514.60	nd	1010.07	1009.45	757.80	757.90	-	-
		FA3G3S3	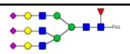	6	5	1	3	0	0	0	-	-	1623.13	nd	1082.42	1081.81	812.07	811.59	-	-
		FA4G4S4(Ac)2	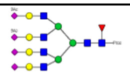	7	6	1	2	2	0	0	-	-	-	-	1329.17	1329.20	997.13	997.22	-	-
		FA4G4S3Ac	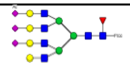	7	6	1	2	1	0	0	-	-	1826.70	nd	1218.14	1218.14	913.85	913.26	-	-
22	6.19	FA4G4S2 or FA3G3S2(LacNAc)1	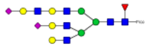	7	6	1	2	0	0	0	-	-	1660.15	nd	1107.10	1106.21	830.58	830.21	-	-
		FA4G4S4	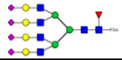	7	6	1	3	1	0	0	-	-	-	-	1315.22	1314.89	986.67	986.53	-	-
23	11.24	FA4G4S3 or FA3G3S3(LacNAc)1	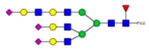	7	6	1	3	0	0	0	-	-	-	-	1204.13	1203.51	903.59	903.73	-	-
		FA4G4S4Ac	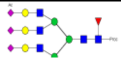	7	6	1	3	1	0	0	-	-	-	-	1315.22	nd	986.67	986.17	-	-

			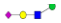																	
**24**	**12.96**	**FA4G4S4**	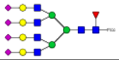	**7**	**6**	**1**	**4**	**0**	**0**	**0**	-	-	-	-	1301.16	1301.18	976.20	976.46	-	-
**25,26**	**2.78**	**FA4G4S2(LacNac)1**	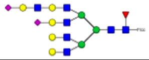	**8**	**7**	**1**	**2**	**0**	**0**	**0**	-	-	1842.71	nd	1228.81	1228.47	921.86	921.44	-	-
**27**	**9.83**	**FA4G4S3(LacNac)1**	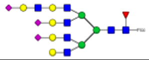	**8**	**7**	**1**	**3**	**0**	**0**	**0**	-	-	-	-	1325.84	1325.90	994.63	995.09	-	-
**28**	**1.31**	**FA4G4S3(LacNac)1**	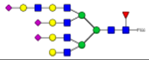	**8**	**7**	**1**	**3**	**0**	**0**	**0**	-	-	-	-	1325.84	nd	994.63	994.24	-	-
		**FA4G4S3Ac2(LacNac)1**	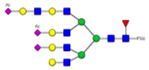	**8**	**7**	**1**	**1**	**2**	**0**	**0**		-	-	-	-	-	1015.64	1014.73	812.71	nd
**29**	**13.67**	**FA4G4S4(LacNac)1**	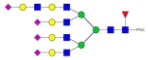	**7**	**6**	**1**	**4**	**0**	**0**	**0**	-	-	-	-	1422.87	1422.56	1067.41	1067.97	854.13	854.19
**30**	**1.76**	**FA4G4S2(LacNac)2**	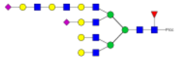	**7**	**6**	**1**	**2**	**0**	**0**	**0**	-	-	-	-	1350.52	nd	1013.14	1012.76	810.72	810.80
**31**	**5.50**	**FA4G4S3(LacNAc)2**	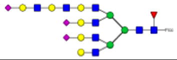	**9**	**8**	**1**	**3**	**0**	**0**	**0**	-	-	-	-	1447.55	nd	1085.96	1085.53	-	-
**32**	**8.26**	**FA4G4S4(LacNAc)2**	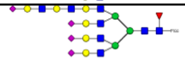	**9**	**8**	**1**	**4**	**0**	**0**	**0**	-	-	-	-	1544.58	nd	1158.69	1158.81	927.15	927.54
**34**	**1.33**	**FA4G4S3(LacNAc)3**	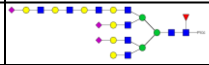	**10**	**9**	**1**	**3**	**0**	**0**	**0**	-	-	-	-	1569.26	nd	1177.20	1176.94	941.96	941.69
**35**	**1.72**	**FA4G4S4(LacNac)3**	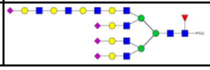	**10**	**9**	**1**	**4**	**0**	**0**	**0**	-	-	-	-	-	-	1249.97	1250.06	1000.20	1000.32
